# Applications of Multi-omics Approaches for Exploring the Molecular Mechanism of Ovarian Carcinogenesis

**DOI:** 10.3389/fonc.2021.745808

**Published:** 2021-09-24

**Authors:** Miaomiao Ye, Yibin Lin, Shuya Pan, Zhi-wei Wang, Xueqiong Zhu

**Affiliations:** Center of Uterine Cancer Diagnosis & Therapy Research of Zhejiang Province, Department of Obstetrics and Gynecology, the Second Affiliated Hospital of Wenzhou Medical University, Wenzhou, China

**Keywords:** ovarian cancer, systems biology, genomics, transcriptomics, proteomics, metabolomics, multi-omics

## Abstract

Ovarian cancer ranks as the fifth most common cause of cancer-related death in females. The molecular mechanisms of ovarian carcinogenesis need to be explored in order to identify effective clinical therapies for ovarian cancer. Recently, multi-omics approaches have been applied to determine the mechanisms of ovarian oncogenesis at genomics (DNA), transcriptomics (RNA), proteomics (proteins), and metabolomics (metabolites) levels. Multi-omics approaches can identify some diagnostic and prognostic biomarkers and therapeutic targets for ovarian cancer, and these molecular signatures are beneficial for clarifying the development and progression of ovarian cancer. Moreover, the discovery of molecular signatures and targeted therapy strategies could noticeably improve the prognosis of ovarian cancer patients.

## Introduction

Ovarian cancer (OC) ranks as the fifth most common cause of cancer-related death in females, and the American Cancer Society predicts that approximately 21,410 new women will be diagnosed with OC, and that 13,770 women will die from OC in the United States in 2021 (1). OC is generally divided into three major pathological subtypes: epithelial-stromal, germ cell, and sex cord-stromal ovarian cancers; epithelial ovarian cancer (EOC) accounts for 90% of OC cases (2). Unfortunately, OC is generally considered a “silent killer” because of the lack of specific symptoms of OC in patients at the early stage and the lack of effective screening strategies. Therefore, more than 60% of OC patients are diagnosed at an advanced stage with extensive invasion and metastasis (3). The standard clinical treatment of EOC comprises of cytoreductive surgery (whenever possible) followed by chemotherapy (4). Currently, platinum-based chemotherapy followed by surgery is a common treatment strategy for OC patients who are not eligible for surgery at presentation (4, 5). While the initial response rate of patients presenting with OC is 60–80%, 70% of advanced-stage OC patients will relapse within 5 years, and many of them acquire drug-resistance (6, 7). At present, serum cancer antigen 125 (CA125) and human epididymis protein 4 (HE4) are extensively used as circulating biomarkers in OC diagnosis and relapse identification (8). However, the sensitivity and specificity of OC diagnosis should be improved, particularly for early-stage OC patients. The failure of early-stage diagnosis and the development of chemoresistance contribute to the high mortality rates of OC patients. Thus, it is necessary to further elucidate the molecular mechanisms of ovarian carcinogenesis and chemoresistance in OC, and to identify effective molecular targets for early-stage diagnosis and clinical treatment. Recently, multi-omics approaches have been applied to explore the mechanisms of OC development, and our article aims to review advancements in OC research from genomic (DNA), transcriptomic (RNA), proteomic (protein), and metabolomic (metabolite) perspectives.

## Multi-Omics Approaches in Ovarian Cancer

Systems biology has been conducted to gain deeper insight into the mechanisms of the physiology and pathophysiology of human health and disease (9), which span multiple areas involving biological sciences, mathematics, engineering, physics, and computer science (10). Systems biology belongs to an interdisciplinary research field, which integrates experimental and computational approaches to investigate the complex biological systems (11). Systems biology is mainly benefited from the functional analysis of large-scale/high-throughput data (12). Many strategies have been implemented to exploit the diverse parameters underlying these large-scale/high-throughput data, such as the inference of gene regulatory networks (GRNs) (13), or machine learning algorithms and Random Forest (RF) algorithm (14), or Gaussian process regression (GPR) (15), or Pathway Inspector (PI) (16), or using mass spectrometry platforms (17). Furthermore, systems biology approaches have brought unprecedented abilities to screen many potential factors (e.g. DNA, RNA, protein, metabolite) and their interaction networks (18).

Systems biology must adopt the data from multi-omics approaches to fully understand the biology of development and progression of diseases *via* using computational and bioinformatics methods and tools. Multi-omics technologies obtain huge datasets that must be analyzed by biological scientists to generate the required information regarding biological systems, which are integral part of systems biology. In the last two decades, researchers have applied various multi-omics approaches to search for novel biomarkers for diagnosis and treatment *via* genomics (19), transcriptomics (20), proteomics (21), and metabolomics (22) studies in diverse human cancers. DNA microarrays are extensively applied for genomics analysis, which are comprised of microscopic spots of DNA oligonucleotides, each with a specific DNA sequence (known as probes), are a multiplex technology and can explore the transcriptional and genomic profiles for thousands of genes (23). Transcriptomics are conducted to figure out the variation of ribonucleic acid (RNA) level in a cell, thus providing detailed and useful information at the transcriptional aspects, including messenger RNAs (mRNAs) microarrays and noncoding RNAs (ncRNAs) microarray, such as microRNAs (miRNA) arrays, long noncoding RNAs (lncRNAs) arrays, circular RNAs (circRNAs) arrays (24). Transcriptomics could be captured and analyzed by RNA microarrays and RNA sequencing (25).

The emergence of proteomics techniques has enabled the large-scale analysis of the full protein components of complex organelles, a single cell, a specific tissue, or biological fluids (26, 27). Proteomics systematic study the structure, function, and interaction of proteins, which is crucial in understanding the molecular mechanisms of diseases comprehensively and aiding at the prevention, diagnosis, and treatment of diseases (28). Researchers have performed Reverse Phase Protein Microarrays (29), Multiplexed Antibody-Based Protein Arrays (30), Proteome Chips (31), Mass Spectrometry (MS) (32), and other techniques in proteomic analysis.

**Figure 1:**
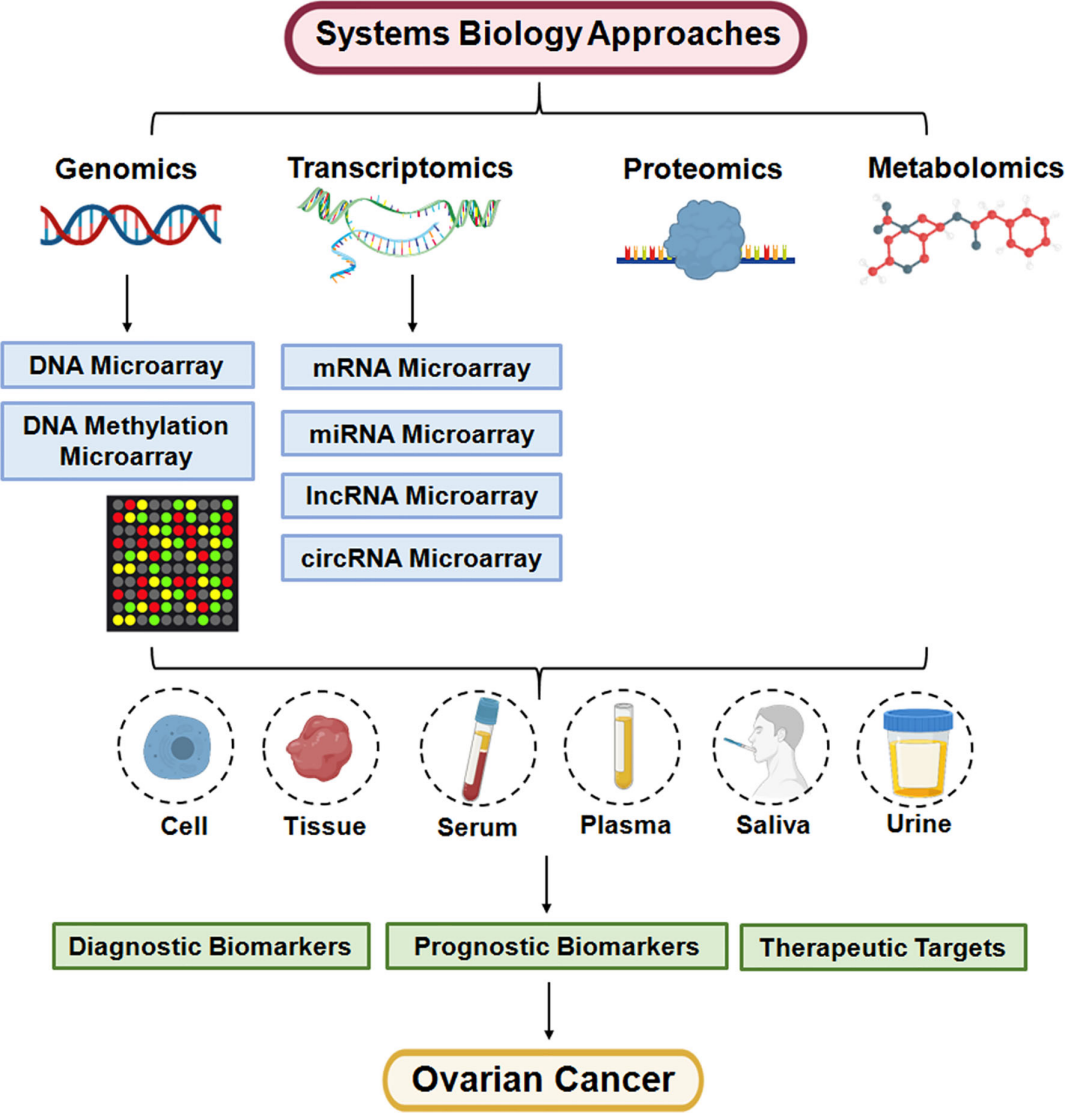
Systems biology approaches are applied for personalized medicine of ovarian cancer.

Metabolomics is considered a high-throughput technology to complement the genotype-phenotype landscape, and can be applied to explore hundreds to thousands of metabolites in biofluids, cells, and tissues (33). Metabolomics is a promising tool for cancer research, and mass spectrometry (MS) and nuclear magnetic resonance (NMR) spectroscopy are commonly used techniques (34). Moreover, multi-omics approaches have been applied in OC and are helpful for improving the diagnosis and prognosis evaluation of OC (**Figure 1**).

### Genomics

Genomics is a technique giving us the ability to investigate the genome-wide structure, function and regulation (35). The application of genomics in OC generally illustrates the regulation of oncogene and antioncogene profiles at the DNA level (**Table 1**). DNA microarrays are used for exploring the genomic profiles *via* a collection of microscopic spots with thousands of probes attached to a solid surface (23). The probes of DNA microarrays are either complementary DNA (cDNA) or shorter oligodeoxynucleotide sequences (74). Moreover, DNA methylation microarrays are employed to assess the epigenetic modifications, which could regulate gene expression with no changes in the nucleotide sequence (75).

**Table 1:** The application of genomics for exploring the candidate biomarkers for ovarian cancer.

Applied Methods	Subjects	Gene Symbol(s)	Ref.
DNA microarray	Tissues	KPNA2	(36)
DNA microarray	Tissues	LGALS4	(37)
DNA microarray	Tissues	SCGB2A1	(38)
DNA microarray	Tissues	FOXM1	(39)
DNA microarray	Cells	ITGB1BP3, COL3A1, COL5A2, COL15A1, TGFBI, DCN, LUM, MATN2, POSTN, EGFL6, ITGA1, COL1A2, LAMA2, GPC3, KRT23, VIT, HMCN1	(40)
DNA microarray	Cells	IGF-1R, HER3	(41)
DNA microarray	Tissues	IGF1/PI3K/NFκB/ERK signaling pathway	(42)
DNA microarray	Tissues	COMT, NLK, HMGI, ErbB-3, S100-α protein, ACBP, COUP-TFII	(43)
DNA microarray	Cells	ARL4C	(44)
DNA microarray	Cells	p53	(45)
DNA microarray	Tissues	COL11A1	(46)
DNA microarray	Tissues	TMPRSS4, MASP1/3, SPC18, PSMB1, IGFBP2, CFI - encoding Complement Factor I, MMP9, ADAM-10	(47)
DNA microarray	Cells	LMX1B	(48)
DNA microarray	Cells	AIFM2, AKTIP, AXIN2, CASP5, FILIP1L, RBBP8, RGC32, RUVBL1, STAG3	(49)
DNA microarray	Cells	ASXL1, H3F3B, CDC73, TGF-beta receptor pathway members, YAP1-MAML2, IKZF2/ERBB4	(50)
DNA microarray	Tissues	PIK3R3	(51)
DNA microarray	Tissues	PKCι, PKCβ1, PKCγ, PKCξ, PKCθ	(52)
DNA microarray	Tissues	RNU6-135P, RNU61262P, VTRNA1-1, MCAM, genes belonging to SNAR gene family	(53)
DNA microarray	Tissues	CREB/ATF, NF-jB/Rel, STAT, Ets family transcription factors	(54)
DNA microarray	Tissues	EGR1, FOSB	(55)
DNA microarray	Cells	EFEMP1	(56)
DNA microarray	Cells	SPARC	(57)
DNA microarray	Tissues	CD9	(58)
DNA microarray	Cells	GLUT-1	(59)
DNA microarray	Tissues	AGR2	(60)
DNA microarray	Cells	CKB	(61)
DNA microarray	Cells	ALDH1A2	(62)
DNA microarray	Cells	ALDH1A1	(63)
DNA microarray	Cells	CCNA1	(64)
DNA microarray	Cells	CDK1	(65)
DNA microarray	Cells	GSK-3α	(66)
DNA microarray	Cells	STAT1	(67)
DNA microarray	Tissues	STAT1, CXCL10, CREB1, MKNK1, MAP3K7, CFL1, PTK2, RIPK1, MYD88, CCL8, CCL7	(68)
DNA Methylation Microarray	Cells	ARNTL	(69)
DNA Methylation Microarray	Tissues	CT45	(70)
DNA Methylation Microarray	Tissues	EGFL7, RASSF1	(71)
DNA Methylation Microarray	Tissues	RUNX3, CAMK2N1	(72)
DNA Methylation Microarray	Cells	ARHGDIB, ARMCX2, COL1A, FLNA, FLNC, MEST, MLH1, NTS, PSMB9	(73)

#### DNA Microarrays

Gene microarray analysis showed that higher expression of karyopherin 2 (KPNA2) was detected in EOC tissues than in human ovarian surface epithelial tissues (36). Moreover, the overexpression of KPNA2 was correlated with an advanced stage, a high histologic grade, and tumor recurrence and predicted a poor prognosis in EOC patients (36). Another study established oligonucleotide microarray analysis in different histological OCs, and showed that galectin 4 (LGALS4) was highly and specifically expressed in mucinous EOC but exhibited lower expression in benign mucinous cysts and borderline (atypical proliferative) tumor (37). One study performed gene expression profiling to point out that mammaglobin b (SCGB2A1) was the most prominent differentially expressed gene in OC of all major histological types (38). Upregulation of 468 genes and downregulation of 994 genes were detected in EOC tissues *versus* normal endometrial (NE) tissues, while 596 upregulated genes and 883 downregulated genes were identified in clear-cell EOC tissues *versus* NE tissues by oligonucleotide microarray analysis (39). Notably, forkhead box M1 (FOXM1) was overexpressed in both epithelial and clear-cell EOC tissues, potentially serving as a negative indicator of non-serous EOC patient outcomes, and promoted cancer progression in all platinum-resistant EOC patients (39). Based on microarray analysis, 10 genes, including integrin beta 1 binding protein 3 (ITGB1BP3), collagen type III alpha 1 (COL3A1), collagen type V alpha 2 (COL5A2), collagen type XV alpha 1 (COL15A1), transforming growth factor beta induced (TGFBI), decorin (DCN), lumican (LUM), matrilin 2 (MATN2), periostin (POSTN) and EGF-like domain multiple 6 (EGFL6), were upregulated, and 7 genes such as intergin subunit alpha 1 (ITGA1), collagen type 1 alpha 2 (COL1A2), laminin subunit alpha 1 (LAMA2), glypican 3 (GPC3), keratin 23 (KRT23), vitrin (VIT) and hemicentin 1 (HMCN1) were downregulated in chemo-resistant sublines compared to chemosensitive OC cells (40). The expression of insulin-like growth factor 1 receptor (IGF-1R) and Erb-B2 receptor tyrosine kinase 3 (ERBB3, also known as HER3) genes was increased in the trastuzumab-resistant OC cell line (SKOV3/T) compared to the parent SKOV3 OC cell lines by microarray analysis (41). In addition, 204 genes were identified as differentially expressed between platinum-resistant and platinum-sensitive OC by microarray analysis; IGF1 was mostly upregulated in platinum-resistant OC (42). Notably, the IGF1, phosphatidylinositol 3-kinase (PI3K), nuclear factor kappa B (NFκB), extracellular signal-regulated kinase (ERK) signalling pathways were associated with chemoresistance in high-grade serous ovarian cancer (HGSOC) (42).

The expression of catechol-O-methyltransferase (COMT), neuroleukin (NLK), high mobility group I proteins (HMGI), ERBB3, S100-α protein and acyl-CoA-binding protein (ACBP) was upregulated, and the expression of chicken ovalbumin upstream promoter transcription factor II (COUP-TFII) was downregulated in OC tissues *versus* normal tissues, as indicated by DNA microarray analysis (43). This study showed that most dysregulated genes in OC were involved in the process of glucose/insulin metabolism (43). Comparative genomic hybridization (CGH) analysis identified 6 genes situated at 2q36.1-37.3 that were downregulated in an OC cell line with high metastatic potential (HO-8910PM), and the downregulation of ADP-ribosylation factor-like 4C (ARL4C) facilitated the migration but had no effect on the proliferation of HO-8910PM cells (44). The whole human genome microarray indicated that mutant p53 positively regulated the integrin β4 and Akt signalling pathways, which facilitated the adhesion of OC cells to mesothelial cells (45). Collagen type XI alpha 1 (COL11A1) was identified as a disease progression-related gene by DNA microarray, and COL11A1 facilitated the progression of OC and indicated a poor prognosis in OC patients (46). CLIP-CHIP microarray analysis revealed that 7 genes, including transmembrance serine protease 4 (TMPRSS4), mannan-binding lectin-associated serine protease 1/3 (MASP1/3), signal peptidase complex 18 (SPC18), proteasome 20S subunit beta 1 (PSMB1), IGF binding protein 2 (IGFBP2), CFI-encoding complement factor I, and matrix metallopeptidase 9 (MMP9), were upregulated, while one gene (ADAM-10) was downregulated in patients with early OC recurrence *versus* those with late or no OC recurrence (47). Moreover, higher expression of ADAM-10 was related to a lower risk of progression, while higher expression of CFI was associated with a higher risk of progression of OC (47). Genome-wide copy number analysis identified a recurrent amplification domain on mouse chromosome 2qB, and the LIM homeobox transcription factor 1 beta (LMX1B) gene was located at this site (48). Notably, LMX1B facilitated the migration of human OC cells and enhanced xenograft growth in nude mice (48). Moreover, global gene expression analysis identified that the NFκB pathway may serve as a mediator of LMX1B-overexpressing OC progression (48). Microcell-mediated chromosome transfer and expression microarray analysis demonstrated that nine genes, including apoptosis inducing factor mitochondria associated 2 (AIFM2), Akt interacting protein (AKTIP), Axin 2 (AXIN2), caspase 5 (CASP5), filamin A interacting protein 1 like (FILIP1L), RB binding protein 8 (RBBP8), response gene to complement 32 (RGC32), RuvB like AAA ATPase 1 (RUVBL1), and stromal antigen 3 (STAG3), were correlated with functional inhibition of OC cell oncogenicity (49). Notably, this study confirmed that a common allele of STAG3 was involved in the development of EOC (49). Genome-wide analyses showed that the amplification or upregulation of additional sex combs like 1 (ASXL1) and hisotone 3 family 3B (H3F3B), deletion or downregulation of CDC73 and transforming growth factor beta (TGF-β) receptor pathway members, and rearrangements of Yes 1 associated transcriptional regulator (YAP1)/mastermind like transcriptional coactivator 2 (MAML2) and IKAROS family zinc finger 2 (IKZF2)/ERBB4 may play a role in the development of ovarian cancer (50). Based on high resolution array comparative genomic hybridization and microarray retrieval approaches, this study indicated that PI3K regulatory subunit 3 (PIK3R3), a member of the PI3K family, had significant DNA copy number gains and that the expression of PIK3R3 mRNA was upregulated in OC compared with normal ovaries (51). Furthermore, the siRNA-induced knockdown of PIK3R3 promoted the apoptosis of OC cells (51). Another study indicated that protein kinase C (PKC) family members (PKCι, PKCβ1, PKCγ, PKCξ, PKCθ) showed significant DNA copy number gains in OC tissues and indicated that the expression of PKCι may play an oncogenic role in human OC (52).

Based on genome-wide transcriptome analysis, in the comparison of primary OC and the peritoneal tumoral implant, the RNU6-135P (RNA, U6 small nuclear 135, pseudogene), RNU61262P, and VTRNA1-1 (Vault RNA) genes were overexpressed in primary OC, while melanoma cell adhesion molecule (MCAM) and genes belonging to the small NF90-associated RNAs (SNAR) gene family were overexpressed in peritoneal tumoral implants (53). When compared primary OC with malignant cells in the ascites, 762 genes were overexpressed in primary OC and 216 genes were overexpressed in malignant cells in ascites (53). Between malignant cells in the ascites and the peritoneal tumoral implant, 515 genes were overexpressed in the peritoneal tumoral implant, and 133 genes were overexpressed in the malignant cells in ascites (53). Notably, this study demonstrated that the ubiquitin-specific protease-17 (USP17) gene family was potentially a target for epithelial-mesenchymal transition (EMT) in HGSOC (53). DNA microarray analyses revealed that 266 human transcripts were aberrantly expressed in OC *versus* normal tissues from patients with elevated biobehavioral risk factors (high depressive symptoms and low social support) with respect to grade- and stage-matched OC from low-risk patients (54). Notably, β-adrenergically-linked transcription control pathways, including cyclic AMP response element binding protein (CREB)/activating transcription factor (ATF), NF-jB/Rel, signal transducer and activator of transcription (STAT), and Ets family transcription factors, were activated in high biobehavioral risk patients (54). Oligonucleotide microarray analysis revealed that 52 candidate genes in the stroma were related to the progression-free survival (PFS) of EOC patients (55). Moreover, the overexpression of the early growth response 1 (EGR1) and FBJ murine osteosarcoma viral oncogene homologue B (FOSB) genes in stromal cells indicated a poor prognosis in EOC patients (55). One study performed whole-genome analysis and found that loss of heterozygosity (LOH) of the 13q domain potentially predicted prolonged PFS in OC patients (76).

The complementary DNA (cDNA) microarray showed that 1596 genes were differentially expressed between OC subclones with low invasive potential and those with high invasive potential (56). Moreover, epidermal growth factor–containing fibulin-like extracellular matrix protein 1, fibulin-3 (EFEMP1) was significantly upregulated in a highly invasive subclone and promoted the invasion and metastasis of OC cells by activating the PI3K/AKT pathway (56). Another study revealed that the expression level of secreted protein acidic and rich in cysteine (SPARC) was higher in highly invasive subclone than in less invasive subclone of OC cells by cDNA microarray analysis; high SPARC expression was correlated with lymph node metastasis, low differentiation, high stage and a poor outcome in OC patients (57). Furthermore, silencing SPARC attenuated the proliferation, invasion and metastasis and promoted the apoptosis of OC cells (57). In addition, overexpression of CD9 was found in borderline and serous-type OC by cDNA microarray profile, and the increased expression of CD9 promoted cell growth by activating the NF-κB pathway (58). A cDNA microarray was performed to display that the expression of glucose transporter-1 (GLUT-1) was elevated in EOC cells compared to normal ovarian cells, and the overexpression of GLUT-1 was associated with a poor outcome in EOC patients (59). Moreover, cDNA microarray analysis indicated that the expression of human anterior gradient 2 (AGR2) was upregulated in OC tissues compared to paired normal ovarian tissues (60). Further study demonstrated that AGR2 potentially served as a biomarker for diagnosing mucinous OC and facilitated the proliferative and migratory ability of OC cells (60). One study demonstrated that creatine kinase B (CKB) was upregulated in OC cells compared with normal ovary surface epithelial cells by cDNA microarray analysis (61). Moreover, 24957 genes were dysregulated between OC cells and normal ovarian cells by cDNA microarray analysis (62). Fifteen ALDH (aldehyde dehydrogenase) isoforms exhibited differential expression patterns; for example, the downregulation of ALDH1A2 (aldehyde dehydrogenase 1 family member A2), ALDH1B1 and ALDH9A1 and the upregulation of ALDH3A1 were observed in OC cells (62). ALDH1A2 was the most significantly downregulated gene, and lower expression of ALDH1A2 was associated with a worse prognosis of OC patients (62). Another study performed oligonucleotide microarray analysis to illustrate that ALDH1A1 was significantly upregulated in paclitaxel- and topotecan-resistant OC cells and potentially contributed to the development of drug resistance in OC (63). cDNA microarray analysis showed that cyclin A1 (CCNA1) was the most highly overexpressed gene in OC cells *versus* normal cells, and was prominently correlated with the paclitaxel-, doxorubicin and 5-fluorouracil -resistance of OC cells (64). Moreover, cyclin-dependent kinase 1 (CDK1) was upregulated in paclitaxel-resistant EOC cells compared to normal EOC cells according to cDNA microarray analysis, and CDK1 served as a target of paclitaxel resistance-related transcription factors (65). Furthermore, elevated expression of glycogen synthase kinase-3α (GSK-3α) was found in paclitaxel-resistant OC cells by cDNA microarray analysis (66). One study conducted cDNA microarray analysis to reveal that the expression of STAT1 was correlated with decreased sensitivity to cisplatin and cis-diamminedichloro (2-methylpyridine) platinum (II) (AMD473) in OC cells (67). Similarly, one group applied a NanoString nCounter platform with a panel of 184 human inflammation genes in 15 chemoresistant and 19 chemosensitive HGSOC (68). This study illustrated that 11 genes, including STAT1, C-X-C motif chemokine ligand 1 (CXCL10), CAMP responsive element binding protein 1 (CREB1), MAPK interacting serine/threonine kinase 1 (MKNK1), mitogen-activated protein kinase kinase kinase 7 (MAP3K7), cofilin 1 (CFL1), protein tyrosine kinase 2 (PTK2), receptor interacting serine/threonine kinase 1 (RIPK1), myeloid differentiation primary response 88 (MYD88), C-C motif chemokine ligand 8 (CCL8) and CCL7, were upregulated in chemosensitive HGSOC *versus* chemoresistant HGSOC, and STAT1 was the most significantly upregulated gene (68). Taken together, DNA microarray technology provides the chance to obtain a genome-wide scale for rapid analysis of mechanism involved in OC initiation and progression.

#### DNA Methylation Microarrays

DNA methylation microarrays can obtain the methylation profile of DNA promoter regions and CpG islands using affinity-based isolation methods, such as methylated DNA immunoprecipitation (MDIP) assay, which will help us understand the molecular mechanism of epigenetic events. One study carried out MDIP-chip analysis in various OC cell lines and clarified that aryl hydrocarbon receptor nuclear translocator-like (ARNTL), which is an HLH-containing transcription factor, was methylated in a subset of OC cell lines (69). The upregulation of ARNTL attenuated the growth and improved the cisplatin-sensitivity of OC cells; however, ARNTL was epigenetically silenced in OC cells (69). One group conducted a DNA methylation microarray in normal ovarian tissues and EOC tissues (70). This report indicated that cancer testis antigen 45 (CT45) was downregulated and hypermethylated in normal ovarian tissues, and was upregulated in EOC tissues concomitant with DNA promoter hypomethylation (70). Another study detected that epidermal growth factor-like 7 (EGFL7) and ras association domain-containing protein 1 (RASSF1) exhibited prominently higher promoter methylation in EOC tissues than in benign ovarian tissues according to DNA methylation array (71). Genome-wide methylation analysis revealed 106 hypo- and 114 hypermethylated regions in ovarian cancer tissues from patients with a poor prognosis compared to those from patients with a good prognosis (72). Notably, the hypermethylation of RUNX family transcription factor 3 (RUNX3) and calcium/calmodulin-dependent protein kinase II inhibitor 1 (CAMK2N1) was correlated with a detrimental prognosis in EOC patients (72). Moreover, genome-wide DNA methylation analysis showed that 9 genes, including Rho GDP dissociation inhibitor beta (ARHGDIB), armadillo repeat containing X-linked 2 (ARMCX2), COL1A, filamin A (FLNA), filamin C (FLNC), mesoderm specific transcript (MEST), mutL homolog 1 (MLH1), neurotensin (NTS) and proteasome 20S subunit beta 9 (PSMB9), were hypermethylated in OC at relapse following chemotherapy or in chemo-resistant cell lines obtained at the time of patient relapse (73). In addition, 5 genes such as ARMCX2, COL1A1, midkine (MDK), MEST and MLH1) were methylated in drug-resistant ovarian cancer cells (73). In summary, DNA methylation microarrays provide the global alterations of DNA methylation in ovarian oncogenesis and progression, which could help us understand the epigenetic regulation of genes in ovarian tumorigenesis.

### Transcriptomics

Transcriptomics is often used to obtain profiles of the RNA transcripts (transcriptomes) in a specific cell or organism with a specific condition or a specific time, which provides a connection between the genome and proteome. The transcriptome comprises coding mRNAs and ncRNAs, including microRNAs (miRNAs), long noncoding RNAs (lncRNAs), circular RNAs (circRNAs), ribosomal RNAs (rRNAs), and transfer RNAs (tRNAs) (77). Specifically, mRNA arrays, miRNA arrays, lncRNA arrays, and circRNA arrays have been performed to explore the molecular mechanisms of OC (**Table 2**).

**Table 2:** The application of transcriptomics for exploring the candidate biomarkers for ovarian cancer .

Applied Methods	Subjects	RNA Symbol(s)	Ref.
MRNA microarray	Tissues	MUC13	(78)
MRNA microarray	Saliva	AGPAT1, B2M, BASP2, IER3, IL1B	(79)
MRNA microarray	Cells	MMP3, MTA1, FN1, MET, CDH1, TIMP2	(80)
MRNA microarray	Cells	CAMK2D, SMARCA2	(81)
MiRNA microarray	Serum	Has-let−7d−3p	(82)
MiRNA microarray	Serum	miRNAs-21, 92, 93, 126, 29a, 155, 127, 99b	(83)
MiRNA microarray	Serum	miR-486-5p	(84)
MiRNA microarray	Serum	hsa-miR-1273g-3p	(85)
MiRNA microarray	Tissues	miR-196b	(86)
MiRNA microarray	Tissues	miR-551b, miR-19b, miR-196b, miR-3198, miR-8084, miR-3201, miR-3613, miR-7515	(87)
MiRNA microarray	Tissues	miR-199a-3p, miR-199a-5p, miR-181a-5p, let-7g-5p	(88)
MiRNA microarray	Tissues	miR-182	(89)
MiRNA microarray	Serum	miR-132, miR-26a, let-7b, miR-145	(90)
MiRNA microarray	Tissues	miR-129-1−3p, miR−542−5p, miR−450a−5p, miR−129−2−3p, miR-424-5p	(91)
MiRNA microarray	Cells	miR-22	(92)
MiRNA microarray	Tissues	miR-21, miR-125a, miR-125b, miR-100, miR-145, miR-16, miR-99a, miR-200, miR-141, miR-18a, miR-93, and miR-429, let-7b, miR-199a	(93)
MiRNA microarray	Tissues	miR-410, miR-645	(94)
MiRNA microarray	Tissues	miR-337, miR-376b, miR-432, miR-376a, miR-368, miR-495, miR-377, miR-419	(95)
MiRNA microarray	Tissues	miR-1183, miR-126-3p, miR-139-3p, miR-802, miR-23a-5p, miR-23a-3p, miR-802, miR-1234	(96)
MiRNA microarray	Cells	miR-141-3p	(97)
MiRNA microarray	Cells	miR-363, miR-29a,	(98)
MiRNA microarray	Cells	miR-335-5p	(99)
MiRNA microarray	Cells	miR-130a	(100)
MiRNA microarray	Cells	miR-30c, miR-130a, miR-335	(101)
MiRNA microarray	Tissues	miR-9, miR-640	(102)
MiRNA microarray	Cells	miR-17~92	(103)
MiRNA microarray	Cells	miR-106a, miR-591	(104)
MiRNA microarray	Tissues	miRNA-1307	(105)
MiRNA microarray	Tissues	miRNA let-7i	(106)
MiRNA microarray	Cells	miR-21	(107)
MiRNA microarray	Cells	miR-129b-1-3p, miR-139-5p, miR-1290, miR-3131	(108)
MiRNA microarray	Cells	miR-99a-5p	(109)
LncRNA microarray	Cells	lncRNA NPBWR1-2	(110)
LncRNA microarray	Tissues	lncRNA HCP5	(111)
LncRNA microarray	Tissues	Linc00152	(112)
LncRNA microarray	Tissues	BC041954, ENST00000423200, uc.428+, BC028018, ENST00000433201, ENST00000458624, ENST00000453838, CR601061, ENST00000505048, ENST00000502715, AK123324, AF087976, NR_001284, ENST00000474313, AL832916, AF086261, BC070168, uc001zfv.1, NR_023313, uc002btm.2	(113)
LncRNA microarray	Tissues	lncRNA RHPN1-AS1	(114)
LncRNA microarray	Tissues	HMGA1P6	(115)
LncRNA microarray	Tissues	lncRNA SOCAR	(116)
LncRNA microarray	Tissues	lncRNA MIAT	(117)
LncRNA microarray	Tissues	lncRNA CTD-2020K17.1	(118)
LncRNA microarray	Cells	lncRNA MALAT1, H19, UCA1, CCAT1, LOC645249, LOC100128881, LOC100292680	(119)
LncRNA microarray	Cells	lncRNA TC0101441	(120)
LncRNA microarray	Cells	LINC01118	(121)
LncRNA microarray	Cells	LncRNA UCA1	(122)
LncRNA microarray	Tissues	LncRNA UCA1	(123)
LncRNA microarray	Tissues	lncRNA linc00161	(124)
LncRNA microarray	Cells	lncRNA ENST00000457645	(125)
LncRNA microarray	Tissues	lncRNA GAS5	(126)
CircRNA microarray	Tissues	circRNA1656	(127)
CircRNA microarray	Tissues	circEXOC6B, circ-N4BP2L2	(128)
CircRNA microarray	Tissues	hsa_circ_0063809	(129)
CircRNA microarray	Tissues	circRNA Cdr1as	(130)

#### mRNA Microarrays

It is known that mRNA microarrays are used to obtain a comprehensive view of changes in mRNA expression patterns *via* a molecular hybridization between oligonucleotide probes and fragments of complimentary mRNA from the interested samples. Analysis of one mRNA microarray indicated that 444 genes were upregulated and 529 genes were downregulated; the expression of mucin 13 (MUC13) mRNA was significantly elevated in metastatic implants from ovarian cancer xenografts *versus* ovarian cancer cells (78). Moreover, MUC13 promoter regions were hypomethylated in OC xenografts, and the overexpression of MUC13 enhanced the migratory and invasive abilities of OC cells (78). One study conducted salivary mRNA microarray analysis of samples from OC patients and healthy controls, and the expression of the 1-acylglycerol-3-phosphate O-acyltransferase 1 (AGPAT1), beta-2-microglobulin (B2M), brain acid soluble protein 2 (BASP2), immediate early response 3 (IER3), and interleukin 1 beta (IL1B) mRNA biomarkers was downregulated in the saliva of OC patients (79). Moreover, the combination of these five mRNA biomarkers in saliva could be used to distinguish OC patients from healthy controls (79). When the lncRNA antisense noncoding RNA in the INK4 locus (ANRIL) was silenced by siRNA in a highly metastatic SOC cell line (SK-OV-3.ip1), four downregulated genes (MMP3, metastasis associated 1 (MTA1), fibronectin 1 (FN1) and MET) and two upregulated genes, including CDH1 and TIMP metallopeptidase inhibitor 2 (TIMP2) genes were detected through tumor metastasis-related mRNA microarray analysis (80). Moreover, the mRNA expression levels of CAMK IIδ (CAMK2D) and SWI/SNF-related matrix-associated actin-dependent regulator of chromatin subfamily A member 2 (SMARCA2) were elevated in cisplatin-resistant OC cells, as detected by next-generation sequencing, and this change was confirmed to enhance the cisplatin resistance of OC cells (81). Clearly, mRNA expression patterns from mRNA microarray in ovarian tumor tissues are pivotal in exploring the underlying mechanisms of ovarian oncogenesis. However, the alteration of mRNAs need to be validated by other methods, such as RT-PCR.

#### MiRNA Microarrays

MiRNAs are a type of small, single-stranded noncoding RNAs and miRNA microarrays are utilized to obtain the changes in the miRNA expression profile on a global scale, which can analyze the differential miRNA expression between tumors and normal tissues. The aberrant expression of distinct miRNAs is associated with several malignancies, including OC (131). Based on the profiling of circulating miRNA/small nuclear RNA (snRNA), higher expression of 6 miRNAs and U2-1 snRNA fragment (RNU2-1f), and lower expression of 16 miRNAs were found in the serum of OC patients than in the serum of healthy controls (132). Moreover, snRNA RNU2-1f abundance dynamics were beneficial for predicting the high risk of recurrence and a detrimental outcome in OC patients following adjuvant chemotherapy (132). In addition, 31 miRNAs were differentially expressed between the serum of EOC patients and that of healthy patients, which was confirmed by miRNA microarray analysis (82). Moreover, the miRNA hsa-let-7d-3p was downregulated in EOC patients, and ROC curve analysis suggested that hsa-let-7d-3p could discriminate EOC patients from healthy patients (82). The combination of miRNA microarray with real-time PCR revealed that miRNA-21, 92, 93, 126 and 29a were significantly upregulated and that miRNA-155, 127 and 99b were downregulated in the serum from OC patients *versus* that of healthy controls (83). MiRNA microarray analysis was performed to assess the serum of patients with ovarian endometrioma and endometriosis-associated OC, and showed that 51 miRNAs were dysregulated (84). MiR-486-5p was upregulated and promoted the proliferation and migration of endometriosis-associated OC cells (84). Moreover, miRNA microarray analysis indicated that hsa-miR-1273g-3p was downregulated in recurrent EOC serum samples *versus* healthy control serum samples (85). The circulating hsa-miR-1273g-3p level could potentially distinguish recurrent EOC patients from healthy controls (85). Another study demonstrated that higher expression of 33 miRNAs and lower expression of 18 miRNAs were found in recurrent EOC than in primary EOC by miRNA microarray (86). Furthermore, the upregulation of miR-196b facilitated the invasion of recurrent EOC by targeting homeobox A9 (HOXA9) (86). The miRNA microarray analysis showed that miR-551b, miR-19b, miR-196b and miR-3198 were significantly upregulated and that miR-8084, miR-3201, miR-3613 and miR-7515 were significantly downregulated in recurrent EOC tissues *versus* primary EOC tissues (87). One study showed that 369 miRNAs were dysregulated between matched OC tissues obtained at an initial laparoscopic evaluation and interval-debulking surgery (IDS) after four cycles of platinum-based chemotherapy by miRNA microarray, which can be classified into five families: miR-199, let-7, miR-30, miR-181 and miR-29 (88). Moreover, the expression levels of miR-199a-3p, miR-199a-5p, miR-181a-5p and let-7g-5p were related to overall survival (OS) and PFS, while those of miR-199a-3p, miR-199a-5p and miR-181a-5p were correlated with residual tumor volume and platinum-free interval of OC patients (88). Moreover, 164 miRNAs were upregulated and 194 miRNAs were downregulated in EOC tissues compared with normal ovarian tissues by miRNA microarray (89). This report also indicated that higher expression of miR-182 was associated with a shorter OS of EOC patients (89). Frequent copy number gains in the sequences mapping for miR-182 within 7q32.2 were related to the overexpression of miR-182 in EOC tissues, which was demonstrated by array-based comparative genomic hybridization (aCGH) analysis (89). Microarray methylation analysis showed that methylation of the miR-182 promoter was correlated with the downregulation of miR-182 in EOC tissues (89).

Lower expression of 95 miRNAs and higher expression of 88 miRNAs were detected in the serum, tissue, and ascites of OC patients than in those of healthy patients by miRNA microarray analysis (90). Moreover, the downregulation of serum miR-132, miR-26a, let-7b and miR-145 could act as biomarkers for SOC (90). Downregulation of 63 miRNAs and upregulation of 43 miRNAs were detected in SOC tissues *versus* normal oviduct tissues by miRNA microarray (91). Among these dysregulated miRNAs, miR-129-1-3p, miR-542-5p, miR-450a-5p, miR-129-2-3p and miR-424-5p were significantly downregulated in SOC tissues (91). The miRNA microarray profile illustrated that lower expression of miR-22 was found in highly metastatic human SOC SKOV-3ip cells than in less metastatic human SOC SKOV-3 cells and demonstrated that miR-22 suppressed the migratory and invasive abilities of OC cells (92). Based on a miRNA microarray, the expression of miR-21, miR-125a, miR-125b, miR-100, miR-145, miR-16, and miR-99a was aberrantly expressed in SOC compared to normal ovarian tissues (93). Moreover, the upregulation of miR-200, miR-141, miR-18a, miR-93, and miR-429 and the downregulation of let-7b and miR-199a were associated with a poor clinical outcome of SOC patients (93). In addition, microRNA microarray hybridization indicated that the miRNA survival signature (MiSS) comprising miR-410 and miR-645 predicted poor OS in advanced SOC patients (94). Eight miRNAs situated on the chromosome 14 miRNA cluster (Dlk1-Gtl2 region) could act as tumor suppressor genes in EOC, which was confirmed by miRNA microarray, aCGH, cDNA microarray and tissue array analyses (95).

By applying miRNA microarray and multivariate analysis approaches, Ahmad et al. found that miR-1183 and miR-126-3p were correlated with OS; miR-139-3p and miR-802 were associated with the time to progression; and miR-23a-5p, miR-23a-3p and miR-802 were related to the PFS; and miR-1234 was associated with the chemotherapy resistance of EOC (96). Four miRNAs were downregulated and 13 miRNAs were upregulated in COC1/DDP (platinum-resistant) OC cells *versus* COC1 (platinum-sensitive) cells; miR-141-3p was the most upregulated miRNA (97). In addition, miRNA microarray analysis was performed to explore the miRNA expression changes in cisplatin (CIS)-, topotecan (TOP)-, doxorubicin (DOX)- and paclitaxel (PAC)-resistant OC cell lines and demonstrated that 21 miRNAs were upregulated and 19 miRNAs were downregulated in at least one drug-resistant cell line (98). Moreover, this study suggested that these miRNAs targeted key drug resistance genes to exert drug resistance properties in OC (98). For PAC-resistant cell lines, miR-363 inversely regulated the expression of ATP binding cassette subfamily B member 1 (ABCB1) (98). In TOP-resistant cell lines, the downregulation of miR-29a upregulated the expression of collagen type III alpha 1 chain gene (COL3A1) (98). Additionally, 9 aberrantly expressed miRNAs were found between cisplatin-resistant (A2780/DDP) and cisplatin-sensitive (A2780) OC cells by miRNA microarray analysis (99). MiR-335-5p was downregulated in A2780/DDP cells compared with A2780 cells, and the overexpression of miR-335-5p sensitized OC cells to cisplatin by inhibiting the expression of BCL2 like 2 (BCL2L2) (99). Another study reported that higher expression of 24 miRNAs and lower expression of 8 miRNAs were found in A2780/DDP cells than in A2780 cells by miRNA microarray; miR-130a was upregulated in A2780/DDP cells (100). Moreover, miR-130a attenuated the cisplatin sensitivity of A2780 cells by upregulating the expression of MDR1 and phosphatase and tensin homologue located on chromosome 10 (PTEN) (100). Furthermore, the downregulation of miR-30c, miR-130a and miR-335 was found in PAC- and cisplatin-resistant OC cells based on a miRNA microarray, and the activation of the M-CSF gene may contribute to the decrease in miR-130a (101). Increased expression of 16 miRNAs and decreased expression of 23 miRNAs were found in PAC-resistant ST30 OC cells by miRNA microarray (102). Overexpression of miR-9 and miR-640 predicted a favorable prognosis for OC patients, and the mRNA RAB34 was a target of miR-9 (102). Another study indicated that 69 miRNAs were overexpressed and 102 miRNAs were downregulated in PAC-resistant SKOV3-TR30 OC cells by miRNA microarray analysis; the expression of miR-17~92 was upregulated in SKOV3-TR30 cells (103). Moreover, downregulated expression of miR-17~92 contributed to cell cycle arrest in the G2/M phase, suppressed cell growth, and improved the response to PAC by upregulating BCL1-like 11 (BCL2L11, BIM) in ovarian cancer cells (103). Notably, miR-106a was upregulated and miR-591 was downregulated in PAC-resistant SKpac OC cells compared to PAC-sensitive SKOV3 OC cells based on a miRNA microarray (104). Furthermore, the regulation of miR-106a and miR-591 could re-sensitize PAC-resistant cancer cells by promoting apoptosis and suppressing cell migration and proliferation by targeting BCL10, caspase-7, and zinc finger E-box binding homeobox 1 (ZEB1) (104). Using miRNA microarray and gene ontology analysis approaches, this study showed that miRNA-1307 was overexpressed in chemoresistant EOC tissues *versus* chemosensitive counterparts, and the candidate target genes of miR-1307 were involved in nucleotide synthesis and metabolism, cell proliferation and differentiation, and lymphocyte activation (105). The miRNA microarray showed that the expression of miRNA let-7i was downregulated in chemoresistant patients and potentially acted as an indicator of poor PFS in late-stage OC patients (106). Additionally, the expression of miR-21 was elevated in chemoresistant OC cells based on a miRNA microarray, and suppression of miR-21 facilitated apoptosis and ameliorated the chemosensitivity of OC cells (107).

The miRNA microarray analysis exhibited that the 4 miRNAs (miR-129b-1-3p, miR-139-5p, miR-1290, and miR-3131) were more highly expressed in exosomes originating from HGSOC cells (HeyA8 and TYK-nu cell lines) than exosomes originating from normal ovarian epithelial cells (the IOSE cell line) (108). Among these four miRNAs, miR-1290 was the most upregulated and acted as a potential biomarker to discriminate HGSOC patients from OC patients with other histological types (108). Another study reported higher expression of 9 miRNAs (miR-99a-5p, miR-100-5p, miR-125b-1-3p, miR-139-5p, miR-451a, miR-500a-3p, miR-1290, miR-3131, miR-3153) in exosomes derived from HeyA8 and TYK-nu cells than those derived from IOSE cells by exosomal miRNA microarray analysis (109). Moreover, exosomal miR-99a-5p increased the invasive capacity of HGSOC cells by upregulating the expression of fibronectin and vitronectin in neighboring human peritoneal mesothelial cells (109). It is necessary to note that miRNA expression profile on a global scale from the miRNA microarray needs to be further validated due to that it is unclear how each miRNA exerts its function in ovarian cancer development and progression.

#### LncRNA Microarray

Long noncoding RNAs (lncRNAs) are a class of noncoding, endogenous, single-stranded RNAs with a length of more than 22 nucleotides (133). Moreover, lncRNAs can regulate gene expression at different levels *via* mechanisms, including chromatin modification, transcription and post-transcriptional processing and can take part in the modulation of the biological behavior of human cancers (134). LncRNA microarrays are utilized to efficiently screen differential lncRNAs in cancers, which can provide theoretical basis for exploring the molecular mechanisms of tumorigenesis. One group used a lncRNA microarray approach and showed the downregulation of 699 lncRNAs and upregulation of 110 lncRNAs in OC cells compared with ovarian epithelial cells (110). LncRNA neuropeptides B and W receptor 1-2 (NPBWR1-2) was downregulated more than two-fold in OC cells, and vector-mediated NPBWR1-2 overexpression decreased cell viability, inhibited the proliferative, migratory and invasive ability, and facilitated the apoptosis of OC cells by targeting multiple miRNAs (110). Furthermore, overexpression of lncRNA HCP5 was detected in OC by lncRNA microarray (111). Downregulation of lncRNA HCP5 attenuated the proliferative, invasive, migratory abilities of OC and inhibited the EMT process, which might occur through miR-525-5p/PRC1 (polycomb repressive complex 1) crosstalk and the Wnt/β-catenin signalling pathway (111). Additionally, higher expression levels of 9 lncRNAs and lower expression levels of 5 lncRNAs were found in OC tissues than in their normal counterparts, which was confirmed by lncRNA microarray analysis (112). Among these dysregulated lncRNAs, Linc00152 was upregulated in OC and silencing Linc00152 attenuated OC cell proliferation and promoted cell cycle arrest (112). Another study demonstrated that 795 lncRNAs were upregulated and 2075 lncRNAs were downregulated in OC tissues compared with normal ovarian tissues (113). The top 10 most overexpressed lncRNAs in OC were BC041954, ENST00000423200, uc.428+, BC028018, ENST00000433201, ENST00000458624, ENST00000453838, CR601061, ENST00000505048 and ENST00000502715, while the top 10 most decreased lncRNAs in OC were AK123324, AF087976, NR_001284, ENST00000474313, AL832916, AF086261, BC070168, uc001zfv.1, NR_023313 and uc002btm.2 (113). These dysregulated lncRNAs can be categorized into four types: Rinn lincRNAs, HOX clusters, long-intergenic non-coding RNAs (lincRNAs) near coding genes and enhancer lncRNAs near coding genes (113). A total of 326 dysregulated lncRNAs were detected in EOC *versus* para-cancerous control tissues by lncRNA microarray analysis (114). Among these lncRNAs, the lncRNA RHPN1-AS1 was overexpressed and facilitated the carcinogenesis and metastasis of EOC by serving as a ceRNA to sponge miR-596 and activating leucine zipper/EF hand-containing transmembrane-1 (LETM1) expression and the FAK/PI3K/Akt pathway (114). One report demonstrated that 577 pseudogenes were dysregulated in HGSOC *versus* normal fallopian tubes by lncRNA microarray analysis, among which 538 pseudogenes were upregulated (115). High mobility group AT-hook 1 pseudogene 6 (HMGA1P6) was one of the upregulated pseudogenes and exerted an oncogenic role in OC by serving as a competitive endogenous RNA, contributing to a shorter overall survival in OC patients (115).

A lncRNA microarray was applied and illustrated that 37 lncRNAs were upregulated and 22 lncRNAs were downregulated in omental metastasis tissues (OMTs) *versus* paired primary OC tissues (POCTs) (116). This study also indicated that the upregulation of SOCAR, which is a novel OC metastasis-related lncRNA that facilitates the proliferative, migratory and invasive abilities of OC cells by elevating the expression of matrix metallopeptidase 9 (MMP9) through activation of the Wnt/β-catenin signalling pathway (116). Moreover, the expression of myocardial infarction-associated transcript (MIAT) was increased, and the expression of small nucleolar RNA, C/D Box 114 cluster (SNORD114) family members SNORD114-10, SNORD114-2 and SNORD114-11 was decreased in OMTs compared to matched POCTs by lncRNA microarray analysis (117). The lncRNA microarray showed that higher expression of 37 lncRNAs and lower expression of 22 lncRNAs were found in OMTs than in paired POCTs (118). Among these aberrantly expressed lncRNAs, the lncRNA CTD-2020K17.1 was overexpressed in OMTs and facilitated the migratory, invasive, and proliferative abilities of serous OC cells (118). The lncRNA microarray demonstrated that 583 lncRNAs were upregulated and 578 lncRNAs were downregulated in SKOV-3ip cells compared toparental SKOV3 cells; lncRNAs metastasis-associated lung adenocarcinoma transcript 1 (MALAT1), H19, urothelial cancer associated 1 (UCA1), colon cancer-associated transcript 1 (CCAT1), LOC645249, LOC100128881, and LOC100292680 were downregulated in SKOV-3ip cells (119). Estrogen (E2) aberrantly regulated the expression of 115 lncRNAs in E2 receptor (ER) alpha (ERα)-positive EOC cells compared to E2-untreated controls according to lncRNA microarray analysis (120). Furthermore, E2-mediated upregulation of lncRNA TC0101441 promoted the migration and invasion of ERα-positive EOC cells by regulating the expression of MMP2 and MMP3 (120).

Similarly, 40830 dysregulated lncRNAs were found between PAC-resistant OC cells and PAC-sensitive OC cells by lncRNA microarray analysis (121). Furthermore, LINC01118 was upregulated in PAC-resistant OC cells, and promoted PAC resistance, invasion and migration while attenuating apoptosis in EOC cells through modulating the miR-134/ATP binding cassette C1 (ABCC1) axis (121). Based on lncRNA microarray analysis, one study revealed that lncRNA UCA1 was upregulated in PAC-resistant OC cells *versus* PAC-sensitive OC cells and enhanced the resistance of OC to PAC (122). In addition, upregulation of lncRNA UCA1 was observed in cisplatin-resistant OC samples compared to cisplatin-sensitive OC samples by lncRNA microarray analysis (123). Moreover, the lncRNA UCA1 negatively regulated miR-143 and subsequently modulated the expression of FOSL2, which enhanced the cisplatin resistance of OC (123). Another group indicated that lncRNA linc00161 exhibited higher expression in cisplatin-resistant OC than in cisplatin-sensitive OC by lncRNA microarray analysis (124). Notably, linc00161 downregulated the expression of microRNA-128 and subsequently upregulated the expression of mitogen-activated protein kinase 1 (MAPK1), which promoted cisplatin resistance in OC cells (124). One lncRNA microarray demonstrated that 1033 lncRNAs were upregulated and 869 lncRNAs, including lncRNA ENST00000457645, were downregulated in A2780 cells *versus* cisplatin-resistant CP70 OC cells (125). This study also indicated that lncRNA ENST00000457645 decreased the viability and migratory ability of cisplatin-resistant OC cells, suggesting that lncRNA ENST00000457645 could reserve cisplatin resistance in CP70 cells (125). In addition, lower expression of lncRNA growth arrest-specific transcript 5 (GAS5) was found in EOC tissues than in normal ovarian tissues by lncRNA microarray analysis (126). Moreover, upregulated expression of lncRNA GAS5 could improve the sensitivity of OC to cisplatin by reducing the expression of poly (ADP-ribose) polymerase1 (PARP1) by recruiting transcription factor E2F4 to its promoter and subsequently modulating the MAPK signalling pathway (126). It is required to further investigate which lncRNAs obtained from lncRNA microarray analysis are more important in ovarian tumorigenesis.

#### CircRNA Microarrays

Circular RNAs (circRNAs) are a new subtype of regulatory noncoding RNA (ncRNA) molecules that are characterized by covalently closed-loop structures without 5’ caps or 3’ polyadenylated tails (135). Moreover, the stable structure, tissue- and/or development-specific expression patterns, and good conservation are the major properties of circRNAs (136). CircRNA microarrays can provide genome-wide circRNA expression profiles between tumor specimens and normal tissues. High-throughput sequencing of circRNAs indicated increased expression of 354 circRNAs and decreased expression of 356 circRNAs in HGSOC tissues compared to normal ovarian tissues (127). Among these dysregulated circRNAs, circRNA1656 was downregulated and the expression of circRNA1656 was associated with the FIGO stages of OC patients (127). In addition, circRNA sequencing-based circRNA expression profiles showed that 2556 circRNAs were upregulated and 1832 circRNAs were downregulated in EOC tissues compared with normal ovarian tissues (128). Higher expression of circEXOC6B and circ-N4BP2L2 indicated a better prognosis in EOC patients (128). CircRNA microarray analysis was also performed in PAC-sensitive and PAC-resistant OC tissues (129). This study showed that 341 circRNAs were upregulated and 492 circRNAs were downregulated with fold change ≥ 2.0, and the length of most circRNAs was less than 1500 bp (129). Among these dysregulated circRNAs, hsa_circ_0063809, hsa_circ_0001946, hsa_circ_0026134, hsa_circ_0025033, and hsa_circ_0014130 were the five most upregulated circRNAs (129). In particular, the suppression of hsa_circ_0063809 can reverse PAC resistance in OC cells (129). Upregulation of 148 circRNAs and downregulation of 191 circRNAs between cisplatin-sensitive and cisplatin-resistant OC tissues were observed by circRNA microarray analysis (130). Among these circRNAs, Cdr1as was downregulated in cisplatin-resistant OC, and Cdr1as sensitized OC to cisplatin by suppressing miR-1270 expression and subsequently upregulating suppressor of cancer cell invasion (SCAI) expression (130). In conclusion, a genome-wide circRNA expression profile is *via* lncRNA microarray could facilitate the understanding of ovarian oncogenesis and drug resistance.

### Proteomics

Proteomics techniques can provide the whole proteome or all proteins from a particular cell, tissue, biofluid or organism. Protein expression profiling by proteomics facilitates the identification of potential biomarkers for disease diagnosis and prognosis prediction. Moreover, proteomics techniques are widely applied to assess OC carcinogenesis, and a panel of proteins that are considered helpful biomarkers for OC patients has been identified (**Table 3**).

**Table 3:** The application of proteomics for exploring the candidate biomarkers for ovarian cancer.

Applied Methods	Subjects	Protein Symbol(s)	Ref.
LC-MS/MS	Serum	RBP4	(137)
LTL, iTRAQ	Serum	N-linked sialylated glycopeptides	(138)
MS	Tissues	periostin, thrombospondin	(139)
iTRAQ, MS	Serum	Serotransferrin, Albumin, Hemopexin, C-reactive protein, Amyloid A1	(140)
LC-MS	Tissues	AAT, NFκB, PMVK, VAP1, FABP4, PF4	(141)
2-DE, MALDI-TOF MS	Cells	ACTB, TIM, PDIA3, PDIA1, DCTN2, KIC17, SIAS, KIC10, KIC18, GRP78, CAPG, PPIA, ROA2, LMNA, EZRI, ADRM1, ENOA	(142)
MALDI-TOF/TOF, MS/MS	Cells	UBC13	(143)
iTRAQ, LC-MS/MS	Tissues	Plxdc2, CK7	(144)
MP, MAC	Cells	palmitoylprotein thioesterase 1 precursor, triose phosphate isomerase, ER-associated DNAJ, tumor rejection antigen (gp96) 1	(145)
LC-MS/MS	Cells	AKAP12	(146)
RPPA	Tissues	PDGFRβ, VEGFR2	(147)
2-DE, MALDI-TOF-MS	Serum	haptoglobin proteins, transthyretin, apolipoprotein E, alpha-1-antitrypsin, clusterin, carbonic anhydrase 1	(148)
2-DE	Tissues	stress-70 protein, elongation factor Tu, PRDX2, G3P, GRP75, ENOA, APOA1, EFTU, ANXA	(149)

By employing peptide ligand library beads (PLLB) and 1D gel liquid chromatography tandem mass spectrometry (LC-MS/MS) approaches, one report found that retinol binding protein 4 (RBP4) was highly expressed in the serum of OC patients (137). Lectin-directed tandem labelling (LTL) and isobaric tags for relative and absolute quantitation (iTRAQ) proteomics approaches identified 45 N-linked sialylated glycopeptides comprising 46 glycosylation sites, among which 10 sialylated glycopeptides were overexpressed in the serum of OC patients (138). Moreover, glycoproteomic analysis was performed in endometrioid OC tissues and normal ovarian tissues, and periostin and thrombospondin were confirmed as candidate biomarkers with tumor-specific glycosylation in endometrioid OC patients (139). In addition, iTRAQ-tagging and mass spectrometry analysis showed that the serum proteins serotransferrin, slbumin, hemopexin, C-reactive protein and amyloid A1 were dysregulated in OC samples compared with benign ovarian tumor samples and healthy control samples (140). Notably, the combination of serum amyloid A1, albumin, serotransferrin, human epididymis protein 4 (HE4) and CA125 elevated the diagnostic capacity for differentiating benign and malignant OC (140). Increased expression of 52 peptides and decreased expression of 52 peptides were detected in the ascites fluid of OC patients compared to that of those with benign gynecological conditions (150). Label-free liquid chromatography-mass spectrometry was performed between favorable prognosis and poor prognosis primary HGSOC specimen and revealed that higher expression of 288 proteins was found in the favorable prognosis cluster, while higher expression of 370 proteins was found in the poor prognosis cluster (141). Additionally, the overexpression of α1-antitrypsin (AAT), NFκB, and phosphomevalonate kinase (PMVK) predicted a favorable PFS, and the overexpression of vascular adhesion protein 1 (VAP1), fatty acid-binding protein 4 (FABP4), and platelet factor 4 (PF4) indicated a poor PFS of HGSOC patients (141). Upregulation of 8 proteins, including actin beta (ACTB), T-cell immunoglobulin mucin (TIM), protein disulfide isomerase A3 (PDIA3), PDIA1, dynactin subunit 2 (DCTN2), KIC17, SIAS, and KIC10) and downregulation of 9 proteins, such as KIC18, G protein-coupled receptor 78 (GRP78), capping actin protein, gelsolin like (CAPG), peptidylprolyl isomerase A (PPIA), replication origin activator 2 (ROA2), lamin A/C (LMNA), EZRI, ADRM1, and ENOA) were detected in vascular endothelial growth factor (VEGF)-treated OC cells compared with normal OC cells through proteomic analysis by two-dimensional electrophoresis (2-DE) (142). These 17 proteins are frequently involved in cell growth and metabolism processes (142).

DIGE quantitative proteomics analysis revealed the downregulation of UBC13 (UBE2N, ubiquitin conjugating enzyme E2 N) in PAC-resistant OC cells, and UBC13 modulated PAC sensitivity through the DNA methyltransferase 1 (DNMT1) checkpoint with forkhead and ring finger domain (CHFR)-aurora kinase A (Aurora A) signalling pathway in OC cells (143). iTRAQ-based proteomic analysis in combination with LC-MS/MS revealed that the expression of plexin domain containing 2 (Plxdc2) and cytokeratin 7 (CK7) proteins was elevated in PAC-resistant OC tissues (144). One study indicated that 47 proteins were upregulated and 309 proteins were downregulated both at more than 1.5-fold quantitative alterations in PAC-resistant OC cells *versus* PAC-sensitive OC cells, through LC-MS/MS label-free quantitative proteomics (151). Most of the 356 identified differential proteins were related to pyruvate metabolism, metabolic pathways, glycolysis/gluconeogenesis, protein processing in the endoplasmic reticulum, regulation of actin cytoskeleton, systemic lupus erythematosus, tight junctions and ribosomes (151). Multiplexed proteomics (MP) technology and multilectin affinity chromatography (MAC) indicated that four glycoproteins (palmitoyl protein thioesterase 1 precursor, triose phosphate isomerase, ER-associated DNAJ and tumor rejection antigen (gp96) 1) were upregulated in PAC-resistant A2780TC1 OC cells compared with A2780 OC cells (145). A kinase (PRKA) anchor protein 12 (AKAP12) was overexpressed in the PAC-resistant HGSOC cell secretome according to proteomic analysis, and the upregulation of AKAP12 indicated a poor prognosis in HGSOC patients (146). Strikingly, 11 signalling pathway proteins were upregulated in platinum-resistant OC compared with platinum-sensitive OC according to reversed-phase protein array (RPPA) analysis; the platelet-derived growth factor receptor beta (PDGFRβ) and VEGF receptor 2 (VEGFR2) proteins were most prominently overexpressed (147). Moreover, higher expression of PDGFRβ was associated with worse progression-free and overall survival, while VEGFR2 expression had no considerable relationship with the OS of OC patients (147). Matrix-assisted laser desorption/ionization time of flight mass spectrometry (MALDI-TOF-MS) indicated that serum haptoglobin proteins, transthyretin and apolipoprotein E were upregulated, while serum alpha-1-antitrypsin, clusterin, and carbonic anhydrase 1 were downregulated in chemo-sensitive EOC patients compared with chemo-resistant EOC patients (148). Proteomics analysis of OC tissues illustrated that stress-70 protein, elongation factor Tu, peroxiredoxin (PRDX2), glyceraldehyde 3-phosphate dehydrogenase (G3P), mitochondrial GRP75, α-enolase (ENOA), apolipoprotein A-1 (APOA1), mitochondrial EFTU and annexin A (ANXA) were considered predictive indicators of drug-resistant OC (149). Taken together, whole proteome profiles in ovarian cancer give us a chance to determine the mechanism of ovarian tumorigenesis. However, the changes of all proteins in ovarian cancer should be validated by other approaches such as western blotting analysis.

### Metabolomics

Metabolomics are utilized to obtain metabolites expression profiles in a specific cell, tissue or biofluid *via* a high-throughput technology, which can help us understand the cellular metabolism. Metabolomics function in identifying and quantifying the alteration of diverse metabolite levels of samples in response to disease status, dietary patterns and pharmaceutical interventions (152). Metabolomics is a promising tool for cancer research, and mass spectrometry (MS) and nuclear magnetic resonance (NMR) spectroscopy are commonly used techniques (34). Metabolite profiling by metabolomics helps researchers to gain deeper insight into the changes and interactions of metabolites related to ovarian cancer biology and could improve the personalized clinical treatment of ovarian cancer patients (**Table 4**).

**Table 4:** The application of metabolomics for exploring the candidate biomarkers for ovarian cancer.

Applied Methods	Subjects	Metabolite Symbol(s)	Ref.
UPLC/Q-TOF MS	Serum	2-piperidinone, 1-heptadecanoylglycerophosphoethanolamine	(153)
Wide spectrum targeted metabolomics	Serum	lysoPC a C16:1, PC aa C32:2, PC aa C34:4, PC aa C 36:6	(154)
GC-MS	Serum	EFA (C16:0), EFA (C18:0), FFA(C16:0)	(155)
HILIC, MS/MS	Serum	combination of serum maltose, maltotriose, raffinose, and mannitol	(156)
UPLC-MS	Serum	CPG	(157)
UPLC-MS	Plasm	piperine, 3-indolepropionic acid, 5-hydroxyindoleacetaldehyde hydroxyphenyllactate	(158)
UPLC/Q-TOF/MS	Plasm	adrenoyl ethanolamide, LysoPCs, LysoPE	(159)
RRLC-MS	Plasm	hydroxyphenyllactic acid, coproporphyrinogen, uric acid, lysine, 3-(3,5-diiodo-4-hydroxyphenyl) lactate, 24,25-hydroxyvitamin D3, carnitine, creatinine, l-beta-aspartyl-l-glutamic acid phosphohydroxypyruvic acid	(160)
UPLC-QTOF/MS	Urine	N4-acetylcytidine, succinic acid, urate-3-ribonucleoside, pseudouridine	(161)
HILIC, RPLC, MS	Urine	homovanillic acid sulfate, phytosphingosine, hippuric acid, pseudouridine	(162)
GC/TOF-MS	Tissues	glucose	(163)

According to ultra-performance liquid chromatography and quadrupole time-of-flight mass spectrometry (UPLC/Q-TOF MS), metabolites, including 2-piperidinone and 1-heptadecanoylglycerophosphoethanolamine, in the serum were closely associated with OC and could potentially serve as biomarkers of OC (153). Wide spectrum targeted metabolomics showed that lipid compounds (lysoPC a C16:1, PC aa C32:2, PC aa C34:4 and PC aa C 36:6) in serum were correlated with OC metabolism and potentially related to the growth and progression of OC (154). Moreover, increased expression of saturated fatty acids and decreased expression of unsaturated fatty acids were detected in the serum of EOC patients compared to that of healthy controls, which was confirmed by the gas chromatography-mass spectrometry (GC-MS) metabolomics approach (155). Moreover, serum esterified fatty acids (EFAs) (C16:0), EFAs (C18:0) and free fatty acids (FFAs) (C16:0) were considered biomarkers for discriminating EOC patients from healthy controls (155). Using hydrophilic interaction liquid chromatography(HILIC) and tandem mass spectrometry, this report integrated serum maltose, maltotriose, raffinose, and mannitol into the panel for differentiating OC patients from benign ovarian tumor patients and healthy patients (156). Ultra performance liquid chromatographic-mass spectrometry (UPLC-MS) showed that the upregulation of serum 27-nor-5β-cholestane-3,7,12,24,25 pentol glucuronide (CPG) could be a predictive indicator for EOC in the early stage (157). Metabolic profiling based on UPLC-MS revealed that plasma piperine, 3-indolepropionic acid, 5-hydroxyindoleacetaldehyde and hydroxyphenyllactate could be used for differentiating EOCs from benign ovarian tumors (BOTs)/uterine fibroids (UFs), and for differentiating early-stage EOCs from late-stage EOCs (158). Additionally, one study explored the metabolomics profiles of plasma samples from early-stage EOC patients and healthy controls by UPLC/Q-TOF MS, and 18 metabolites were dysregulated in early stage EOC (159). Among these metabolites, adrenoyl ethanolamide, lysophospholipids (LysoPCs), LysoPE and one unknown compound were identified as potentially useful for discriminating early-stage EOC patients from healthy controls (159). Global metabolomic profiles in the pre- and post-operative plasma of EOC patients were assessed, and the results identified hydroxyphenyllactic acid, coproporphyrinogen, uric acid, lysine, 3-(3,5-diiodo-4-hydroxyphenyl) lactate, 24,25-hydroxyvitamin D3, carnitine, creatinine, l-beta-aspartyl-l-glutamic acid and phosphohydroxypyruvic acid as predictive biomarkers for the recurrence of EOC, and indicated that the combination of pre- and post-operative serum biomarkers showed the best predictive capacity for the recurrence of EOC (160).

The concentrations of four dysregulated urinary metabolomics markers, including N4-acetylcytidine, succinic acid, urate-3-ribonucleoside, and pseudouridine, showed a trend towards the normal level in the post-operative condition compared with the preoperative condition of EOC patients according to UPLC/Q-TOF MS (161). Based on HILIC and reversed-phase liquid chromatography (RPLC) coupled to mass spectrometry, this study identified five urinary metabolites specific to OC, including homovanillic acid sulfate, phytosphingosine, hippuric acid and pseudouridine, and one unknown component (162). Gas chromatography/time of flight mass spectrometry (GC/TOF-MS)-based metabolomics analysis revealed that a higher concentration of glucose and other metabolites from carbohydrate metabolism were found in AMP-activated protein kinase (AMPK)-negative OC than those in the AMPK-positive OC (163). Based on a genome-scale metabolic model and microarray data, one study demonstrated that cisplatin could not kill resistant OC cells, but it could confer a more vulnerable metabolic condition in the cancer cells (164). Metabolites expression profiles in ovarian cancer could enhance the understanding of cellular metabolism, leading to contribution to combating ovarian cancer *via* targeting tumor cell metabolism.

## Conclusion and Perspective

Multi-omics has been used to discover the biomarkers for ovarian cancer prognosis and therapeutic efficacy. However, several disadvantages of multi-omics must be discussed. For example, several factors of technical, instrumental and computational nature will affect the precision of microarray data. The poor storage of clinical sample in hospitals causes the poor quality of RNA samples, leading to inaccurate data by transcriptomics. Moreover, no standard methodology is available for microarrays so far. It is also difficult to develop standard methods to integrate data obtained from various types of microarrays. Appropriate statistical analyses are necessary to analyze multi-omics approaches.

Through different microarrays, thousands of genes and proteins are changed in ovarian carcinogenesis and promotion. How can we judge which genes and proteins are key drivers to induce ovarian tumorigenesis? Different microarray approaches often obtain inconsistent results, which could be due to biological heterogeneity, different statistical and computational analyses, and target selection criterion, indicating that identified genes and proteins by each microarray method should be validated by other several approaches. Transcriptome profiles need to be validated by other methods, such as RT-PCR assay.

In summary, multi-omics approaches are applied to study the molecular mechanism of the development and progression of OC (**Figure 1**). The combination of genomics, transcriptomics, proteomics and metabolomics is helpful for exploring diagnostic and prognostic biomarkers of OC and for gaining deeper insight into the mechanism of OC chemoresistance. Without a doubt, there is a long way to use multi-omics approaches for personalized therapy in ovarian cancer patients.

## Author Contributions

MY and XZ wrote this manuscript. YL and SP prepared the figures and tables. Z-wW performed data accusation and discussion. All authors contributed to the article and approved the submitted version.

## Funding

This work was supported by the Research Fund for Lin He’s Academician Workstation of New Medicine and Clinical Translation (No.19331105).

## Conflict of Interest

The authors declare that the research was conducted in the absence of any commercial or financial relationships that could be construed as a potential conflict of interest.

## Publisher’s Note

All claims expressed in this article are solely those of the authors and do not necessarily represent those of their affiliated organizations, or those of the publisher, the editors and the reviewers. Any product that may be evaluated in this article, or claim that may be made by its manufacturer, is not guaranteed or endorsed by the publisher.

## References

[1] Siegel RL Miller KD Fuchs HE Jemal A. Cancer Statistics, 2021. CA Cancer J Clin (2021) 71(1):7–33. doi: 10.3322/caac.21654 33433946

[2] Haque A Sait KHW Alam Q Alam MZ Anfinan N Wali AWN. MDR1 Gene Polymorphisms and Its Association With Expression as a Clinical Relevance in Terms of Response to Chemotherapy and Prognosis in Ovarian Cancer. Front Genet (2020) 11:516. doi: 10.3389/fgene.2020.00516 32528530 PMC7264409

[3] Kuo CL Jiang ZY Wang YW Lin TY Huang WL Wu FJ. *In Vivo* Selection Reveals Autophagy Promotes Adaptation of Metastatic Ovarian Cancer Cells to Abdominal Microenvironment. Cancer Sci (2019) 110(10):3204–14. doi: 10.1111/cas.14162 PMC6778661 31385416

[4] Krzystyniak J Ceppi L Dizon DS Birrer MJ. Epithelial Ovarian Cancer: The Molecular Genetics of Epithelial Ovarian Cancer. Ann Oncol (2016) 27 Suppl 1(Suppl 1):i4–i10. doi: 10.1093/annonc/mdw083 27141069 PMC4852274

[5] Lee YJ Kim HS Rim JH Lee JY Nam EJ Kim SW. Germline BRCA, Chemotherapy Response Scores, and Survival in the Neoadjuvant Treatment of Ovarian Cancer. BMC Cancer (2020) 20(1):185. doi: 10.1186/s12885-020-6688-8 32131779 PMC7057666

[6] Palaia I Tomao F Sassu CM Musacchio L Benedetti Panici P. Immunotherapy For Ovarian Cancer: Recent Advances And Combination Therapeutic Approaches. Onco Targets Ther (2020) 13:6109–29. doi: 10.2147/OTT.S205950 PMC7326187 32617007

[7] Jayson GC Kohn EC Kitchener HC Ledermann JA. Ovarian Cancer. Lancet (2014) 384(9951):1376–88. doi: 10.1016/S0140-6736(13)62146-7 24767708

[8] Swiatly A Horala A Hajduk J Matysiak J Nowak-Markwitz E Kokot ZJ. MALDI-TOF-MS Analysis in Discovery and Identification of Serum Proteomic Patterns of Ovarian Cancer. BMC Cancer (2017) 17(1):472. doi: 10.1186/s12885-017-3467-2 28683725 PMC5501370

[9] Irizar H Kanchan K Mathias R Bunyavanich S. Advancing Food Allergy Through Omics Sciences. J Allergy Clin Immunol Pract (2020) 9(1):119–29. doi: 10.1016/j.jaip.2020.07.044 PMC7855623 32777389

[10] Emmert-Streib F Dehmer M. Networks for Systems Biology: Conceptual Connection of Data and Function. IET Syst Biol (2011) 5(3):185–207. doi: 10.1049/iet-syb.2010.0025 21639592

[11] Larsson I Uhlen M Zhang C Mardinoglu A. Genome-Scale Metabolic Modeling of Glioblastoma Reveals Promising Targets for Drug Development. Front Genet (2020) 11:381. doi: 10.3389/fgene.2020.00381 32362913 PMC7181968

[12] Logsdon EA Finley SD Popel AS Mac Gabhann F. A Systems Biology View of Blood Vessel Growth and Remodelling. J Cell Mol Med (2014) 18(8):1491–508. doi: 10.1111/jcmm.12164 PMC4190897 24237862

[13] Grassi A Di Camillo B Ciccarese F Agnusdei V Zanovello P Amadori A. Reconstruction of Gene Regulatory Modules From RNA Silencing of IFN-Alpha Modulators: Experimental Set-Up and Inference Method. BMC Genomics (2016) 17:228. doi: 10.1186/s12864-016-2525-5 26969675 PMC4788926

[14] Touw WG Bayjanov JR Overmars L Backus L Boekhorst J Wels M. Data Mining in the Life Sciences With Random Forest: A Walk in the Park or Lost in the Jungle? Brief Bioinform (2013) 14(3):315–26. doi: 10.1093/bib/bbs034 PMC3659301 22786785

[15] Kirk PD Stumpf MP. Gaussian Process Regression Bootstrapping: Exploring the Effects of Uncertainty in Time Course Data. Bioinformatics (2009) 25(10):1300–6. doi: 10.1093/bioinformatics/btp139 PMC2677737 19289448

[16] Bianco L Riccadonna S Lavezzo E Falda M Formentin E Cavalieri D. Pathway Inspector: A Pathway Based Web Application for RNAseq Analysis of Model and Non-Model Organisms. Bioinformatics (2017) 33(3):453–5. doi: 10.1093/bioinformatics/btw636 PMC5408796 28158604

[17] Burkhardt R Kirsten H Beutner F Holdt LM Gross A Teren A. Integration of Genome-Wide SNP Data and Gene-Expression Profiles Reveals Six Novel Loci and Regulatory Mechanisms for Amino Acids and Acylcarnitines in Whole Blood. PloS Genet (2015) 11(9):e1005510. doi: 10.1371/journal.pgen.1005510 26401656 PMC4581711

[18] Paananen J Fortino V. An Omics Perspective on Drug Target Discovery Platforms. Brief Bioinform (2020) 21(6):1937–53. doi: 10.1093/bib/bbz122 PMC7711264 31774113

[19] Lin C-H Funayama S Peng S-F Kuo C-L Chung J-G. The Ethanol Extraction of Prepared Psoralea Corylifolia Induces Apoptosis and Autophagy and Alteres Genes Expression Assayed by cDNA Microarray in Human Prostate Cancer PC-3 Cells. Environ Toxicol (2018) 33(7):770–88. doi: 10.1002/tox.22564 29667321

[20] Liu HY Lu SR Guo ZH Zhang ZS Ye X Du Q. lncRNA SLC16A1-AS1 as a Novel Prognostic Biomarker in Non-Small Cell Lung Cancer. J Investig Med (2020) 68(1):52–9. doi: 10.1136/jim-2019-001080 PMC6996107 31371390

[21] Ribeiro IP Esteves L Anjo SI Marques F Barroso L Manadas B. Proteomics-Based Predictive Model for the Early Detection of Metastasis and Recurrence in Head and Neck Cancer. Cancer Genomics Proteomics (2020) 17(3):259–69. doi: 10.21873/cgp.20186 PMC7259885 32345667

[22] Choi H-J Jhe Y-L Kim J Lim JY Lee JE Shin M-K. FoxM1-Dependent and Fatty Acid Oxidation-Mediated ROS Modulation Is a Cell-Intrinsic Drug Resistance Mechanism in Cancer Stem-Like Cells. Redox Biol (2020) 36:101589. doi: 10.1016/j.redox.2020.101589 32521504 PMC7286985

[23] Gibriel AAY. Options Available for Labelling Nucleic Acid Samples in DNA Microarray-Based Detection Methods. Brief Funct Genomics (2012) 11(4):311–8. doi: 10.1093/bfgp/els015 22510454

[24] Karkossa I Raps S von Bergen M Schubert K. Systematic Review of Multi-Omics Approaches to Investigate Toxicological Effects in Macrophages. Int J Mol Sci (2020) 21(24):9371. doi: 10.3390/ijms21249371 PMC7764599 33317022

[25] Manzoni C Kia DA Vandrovcova J Hardy J Wood NW Lewis PA. Genome, Transcriptome and Proteome: The Rise of Omics Data and Their Integration in Biomedical Sciences. Brief Bioinform (2018) 19(2):286–302. doi: 10.1093/bib/bbw114 27881428 PMC6018996

[26] Milardi D Grande G Vincenzoni F Pierconti F Pontecorvi A. Proteomics for the Identification of Biomarkers in Testicular Cancer-Review. Front Endocrinol (Lausanne) (2019) 10:462. doi: 10.3389/fendo.2019.00462 31354629 PMC6639829

[27] Moulder R Bhosale SD Goodlett DR Lahesmaa R. Analysis of the Plasma Proteome Using iTRAQ and TMT-Based Isobaric Labeling. Mass Spectrom Rev (2018) 37(5):583–606. doi: 10.1002/mas.21550 29120501

[28] Nandal S Burt T. Integrating Pharmacoproteomics Into Early-Phase Clinical Development: State-Of-the-Art, Challenges, and Recommendations. Int J Mol Sci (2017) 18(2):448. doi: 10.3390/ijms18020448 PMC5343982 28218733

[29] Paweletz CP Charboneau L Bichsel VE Simone NL Chen T Gillespie JW. Reverse Phase Protein Microarrays Which Capture Disease Progression Show Activation of Pro-Survival Pathways at the Cancer Invasion Front. Oncogene (2001) 20(16):1981–9. doi: 10.1038/sj.onc.1204265 11360182

[30] He T. Implementation of Proteomics in Clinical Trials. Proteomics Clin Appl (2019) 13(2):e1800198. doi: 10.1002/prca.201800198 30702805

[31] Zhu H Bilgin M Bangham R Hall D Casamayor A Bertone P. Global Analysis of Protein Activities Using Proteome Chips. Science (2001) 293(5537):2101–5. doi: 10.1126/science.1062191 11474067

[32] Li X Wang W Chen J. Recent Progress in Mass Spectrometry Proteomics for Biomedical Research. Sci China Life Sci (2017) 60(10):1093–113. doi: 10.1007/s11427-017-9175-2 29039124

[33] Kumar A Misra BB. Challenges and Opportunities in Cancer Metabolomics. Proteomics (2019) 19(21-22):e1900042. doi: 10.1002/pmic.201900042 30950571

[34] Cheung PK Ma MH Tse HF Yeung KF Tsang HF Chu MKM. The Applications of Metabolomics in the Molecular Diagnostics of Cancer. Expert Rev Mol Diagn (2019) 19(9):785–93. doi: 10.1080/14737159.2019.1656530 31414918

[35] Davies AC Harris D Banks-Gatenby A Brass A. Problem-Based Learning in Clinical Bioinformatics Education: Does It Help to Create Communities of Practice? PloS Comput Biol (2019) 15(6):e1006746. doi: 10.1371/journal.pcbi.1006746 31246944 PMC6597031

[36] Zheng M Tang L Huang L Ding H Liao W-T Zeng M-S. Overexpression of Karyopherin-2 in Epithelial Ovarian Cancer and Correlation With Poor Prognosis. Obstet Gynecol (2010) 116(4):884–91. doi: 10.1097/AOG.0b013e3181f104ce 20859152

[37] Heinzelmann-Schwarz VA Gardiner-Garden M Henshall SM Scurry JP Scolyer RA Smith AN. A Distinct Molecular Profile Associated With Mucinous Epithelial Ovarian Cancer. Br J Cancer (2006) 94(6):904–13. doi: 10.1038/sj.bjc.6603003 PMC2361366 16508639

[38] Bellone S Tassi R Betti M English D Cocco E Gasparrini S. Mammaglobin B (SCGB2A1) Is a Novel Tumour Antigen Highly Differentially Expressed in All Major Histological Types of Ovarian Cancer: Implications for Ovarian Cancer Immunotherapy. Br J Cancer (2013) 109(2):462–71. doi: 10.1038/bjc.2013.315 PMC3721400 23807163

[39] Tassi RA Todeschini P Siegel ER Calza S Cappella P Ardighieri L. FOXM1 Expression Is Significantly Associated With Chemotherapy Resistance and Adverse Prognosis in Non-Serous Epithelial Ovarian Cancer Patients. J Exp Clin Cancer Res (2017) 36(1):63. doi: 10.1186/s13046-017-0536-y 28482906 PMC5422964

[40] Januchowski R Zawierucha P Ruciński M Zabel M. Microarray-Based Detection and Expression Analysis of Extracellular Matrix Proteins in Drug−Resistant Ovarian Cancer Cell Lines. Oncol Rep (2014) 32(5):1981–90. doi: 10.3892/or.2014.3468 25199881

[41] Jia Y Zhang Y Qiao C Liu G Zhao Q Zhou T. IGF-1R and ErbB3/HER3 Contribute to Enhanced Proliferation and Carcinogenesis in Trastuzumab-Resistant Ovarian Cancer Model. Biochem Biophys Res Commun (2013) 436(4):740–5. doi: 10.1016/j.bbrc.2013.06.030 23792093

[42] Koti M Gooding RJ Nuin P Haslehurst A Crane C Weberpals J. Identification of the IGF1/PI3K/Nfκb/ERK Gene Signalling Networks Associated With Chemotherapy Resistance and Treatment Response in High-Grade Serous Epithelial Ovarian Cancer. BMC Cancer (2013) 13:549. doi: 10.1186/1471-2407-13-549 24237932 PMC3840597

[43] Lee B-C Cha K Avraham S Avraham HK. Microarray Analysis of Differentially Expressed Genes Associated With Human Ovarian Cancer. Int J Oncol (2004) 24(4):847–51. doi: 10.3892/ijo.24.4.847 15010821

[44] Su D Katsaros D Xu S Xu H Gao Y Biglia N. ADP-Ribosylation Factor-Like 4C (ARL4C), a Novel Ovarian Cancer Metastasis Suppressor, Identified by Integrated Genomics. Am J Transl Res (2015) 7(2):242–56. PMC4399089 25901194

[45] Lee J-G Ahn J-H Kim TJ Lee JH Choi J-H. Mutant P53 Promotes Ovarian Cancer Cell Adhesion to Mesothelial Cells *via* Integrin β4 and Akt Signals. Sci Rep (2015) 5:12642. doi: 10.1038/srep12642 26223322 PMC4649895

[46] Wu Y-H Chang T-H Huang Y-F Huang H-D Chou C-Y. COL11A1 Promotes Tumor Progression and Predicts Poor Clinical Outcome in Ovarian Cancer. Oncogene (2014) 33(26):3432–40. doi: 10.1038/onc.2013.307 23934190

[47] Trudel D Avarvarei L-M Orain M Turcotte S Plante M Grégoire J. Proteases and Their Inhibitors as Prognostic Factors for High-Grade Serous Ovarian Cancer. Pathol Res Pract (2019) 215(6):152369. doi: 10.1016/j.prp.2019.02.019 30987833

[48] He L Guo L Vathipadiekal V Sergent PA Growdon WB Engler DA. Identification of LMX1B as a Novel Oncogene in Human Ovarian Cancer. Oncogene (2014) 33(33):4226–35. doi: 10.1038/onc.2013.375 24056967

[49] Notaridou M Quaye L Dafou D Jones C Song H Høgdall E. Common Alleles in Candidate Susceptibility Genes Associated With Risk and Development of Epithelial Ovarian Cancer. Int J Cancer (2011) 128(9):2063–74. doi: 10.1002/ijc.25554 PMC3098608 20635389

[50] Papp E Hallberg D Konecny GE Bruhm DC Adleff V Noë M. Integrated Genomic, Epigenomic, and Expression Analyses of Ovarian Cancer Cell Lines. Cell Rep (2018) 25(9):2617–33. doi: 10.1016/j.celrep.2018.10.096 PMC6481945 30485824

[51] Zhang L Huang J Yang N Greshock J Liang S Hasegawa K. Integrative Genomic Analysis of Phosphatidylinositol 3'-Kinase Family Identifies PIK3R3 as a Potential Therapeutic Target in Epithelial Ovarian Cancer. Clin Cancer Res (2007) 13(18 Pt 1):5314–21. doi: 10.1158/1078-0432.CCR-06-2660 17875760

[52] Zhang L Huang J Yang N Liang S Barchetti A Giannakakis A. Integrative Genomic Analysis of Protein Kinase C (PKC) Family Identifies PKCiota as a Biomarker and Potential Oncogene in Ovarian Carcinoma. Cancer Res (2006) 66(9):4627–35. doi: 10.1158/0008-5472.CAN-05-4527 16651413

[53] Yildirim N Kocal GC Isik Z Saatli B Saygili U Uysal T. Ubiquitin-Proteasome Axis, Especially Ubiquitin-Specific Protease-17 ( USP17) Gene Family, Is a Potential Target for Epithelial-Mesenchymal Transition in High-Grade Serous Ovarian Cancer. Reprod Sci (2019) 26(6):794–805. doi: 10.1177/1933719118799189 30198418

[54] Lutgendorf SK DeGeest K Sung CY Arevalo JM Penedo F Lucci J 3rd. Depression, Social Support, and Beta-Adrenergic Transcription Control in Human Ovarian Cancer. Brain Behav Immun (2009) 23(2):176–83. doi: 10.1016/j.bbi.2008.04.155 PMC2677379 18550328

[55] Kataoka F Tsuda H Arao T Nishimura S Tanaka H Nomura H. EGRI and FOSB Gene Expressions in Cancer Stroma Are Independent Prognostic Indicators for Epithelial Ovarian Cancer Receiving Standard Therapy. Genes Chromosomes Cancer (2012) 51(3):300–12. doi: 10.1002/gcc.21916 22095904

[56] Yin X Fang S Wang M Wang Q Fang R Chen J. EFEMP1 Promotes Ovarian Cancer Cell Growth, Invasion and Metastasis *via* Activated the AKT Pathway. Oncotarget (2016) 7(30):47938–53. doi: 10.18632/oncotarget.10296 PMC5216990 27351229

[57] Chen J Wang M Xi B Xue J He D Zhang J. SPARC Is a Key Regulator of Proliferation, Apoptosis and Invasion in Human Ovarian Cancer. PloS One (2012) 7(8):e42413. doi: 10.1371/journal.pone.0042413 22879971 PMC3411787

[58] Hwang JR Jo K Lee Y Sung B-J Park YW Lee J-H. Upregulation of CD9 in Ovarian Cancer Is Related to the Induction of TNF-α Gene Expression and Constitutive NF-κb Activation. Carcinogenesis (2012) 33(1):77–83. doi: 10.1093/carcin/bgr257 22095071

[59] Cho H Lee YS Kim J Chung J-Y Kim J-H. Overexpression of Glucose Transporter-1 (GLUT-1) Predicts Poor Prognosis in Epithelial Ovarian Cancer. Cancer Invest (2013) 31(9):607–15. doi: 10.3109/07357907.2013.849722 24164300

[60] Park K Chung YJ So H Kim K Park J Oh M. AGR2, a Mucinous Ovarian Cancer Marker, Promotes Cell Proliferation and Migration. Exp Mol Med (2011) 43(2):91–100. doi: 10.3858/emm.2011.43.2.011 21200134 PMC3047197

[61] Huddleston HG Wong K-K Welch WR Berkowitz RS Mok SC. Clinical Applications of Microarray Technology: Creatine Kinase B Is an Up-Regulated Gene in Epithelial Ovarian Cancer and Shows Promise as a Serum Marker. Gynecol Oncol (2005) 96(1):77–83. doi: 10.1016/j.ygyno.2004.08.047 15589584

[62] Choi JA Kwon H Cho H Chung JY Hewitt SM Kim JH. ALDH1A2 Is a Candidate Tumor Suppressor Gene in Ovarian Cancer. Cancers (Basel) (2019) 11(10):1553. doi: 10.3390/cancers11101553 PMC6826427 31615043

[63] Januchowski R Wojtowicz K Sterzyſska K Sosiſska P Andrzejewska M Zawierucha P. Inhibition of ALDH1A1 Activity Decreases Expression of Drug Transporters and Reduces Chemotherapy Resistance in Ovarian Cancer Cell Lines. Int J Biochem Cell Biol (2016) 78:248–59. doi: 10.1016/j.biocel.2016.07.017 27443528

[64] Huang K-C Yang J Ng MC Ng S-K Welch WR Muto MG. Cyclin A1 Expression and Paclitaxel Resistance in Human Ovarian Cancer Cells. Eur J Cancer (2016) 67:152–63. doi: 10.1016/j.ejca.2016.08.007 PMC5080661 27669502

[65] Bae T Weon K-Y Lee J-W Eum K-H Kim S Choi JW. Restoration of Paclitaxel Resistance by CDK1 Intervention in Drug-Resistant Ovarian Cancer. Carcinogenesis (2015) 36(12):1561–71. doi: 10.1093/carcin/bgv140 26442525

[66] Fu Y Hu D Qiu J Xie X Ye F Lu W-G. Overexpression of Glycogen Synthase Kinase-3 in Ovarian Carcinoma Cells With Acquired Paclitaxel Resistance. Int J Gynecol Cancer (2011) 21(3):439–44. doi: 10.1097/IGC.0b013e31820d7366 21436692

[67] Roberts D Schick J Conway S Biade S Laub PB Stevenson JP. Identification of Genes Associated With Platinum Drug Sensitivity and Resistance in Human Ovarian Cancer Cells. Br J Cancer (2005) 92(6):1149–58. doi: 10.1038/sj.bjc.6602447 PMC2361951 15726096

[68] Koti M Siu A Clément I Bidarimath M Turashvili G Edwards A. A Distinct Pre-Existing Inflammatory Tumour Microenvironment Is Associated With Chemotherapy Resistance in High-Grade Serous Epithelial Ovarian Cancer. Br J Cancer (2015) 113(12):1746. doi: 10.1038/bjc.2015.81 PMC4702007 26695556

[69] Yeh C-M Shay J Zeng T-C Chou J-L Huang TH-M Lai H-C. Epigenetic Silencing of ARNTL, a Circadian Gene and Potential Tumor Suppressor in Ovarian Cancer. Int J Oncol (2014) 45(5):2101–7. doi: 10.3892/ijo.2014.2627 25175925

[70] Zhang W Barger CJ Link PA Mhawech-Fauceglia P Miller A Akers SN. DNA Hypomethylation-Mediated Activation of Cancer/Testis Antigen 45 (CT45) Genes Is Associated With Disease Progression and Reduced Survival in Epithelial Ovarian Cancer. Epigenetics (2015) 10(8):736–48. doi: 10.1080/15592294.2015.1062206 PMC4622579 26098711

[71] Rattanapan Y Korkiatsakul V Kongruang A Chareonsirisuthigul T Rerkamnuaychoke B Wongkularb A. EGFL7 and RASSF1 Promoter Hypermethylation in Epithelial Ovarian Cancer. Cancer Genet (2018) 224-225:37–40. doi: 10.1016/j.cancergen.2018.04.117 29778234

[72] Häfner N Steinbach D Jansen L Diebolder H Dürst M Runnebaum IB. RUNX3 and CAMK2N1 Hypermethylation as Prognostic Marker for Epithelial Ovarian Cancer. Int J Cancer (2016) 138(1):217–28. doi: 10.1002/ijc.29690 26175272

[73] Zeller C Dai W Steele NL Siddiq A Walley AJ Wilhelm-Benartzi CSM. Candidate DNA Methylation Drivers of Acquired Cisplatin Resistance in Ovarian Cancer Identified by Methylome and Expression Profiling. Oncogene (2012) 31(42):4567–76. doi: 10.1038/onc.2011.611 22249249

[74] Ramaswamy S Golub TR. DNA Microarrays in Clinical Oncology. J Clin Oncol (2002) 20(7):1932–41. doi: 10.1200/JCO.2002.20.7.1932 11919254

[75] James P Sajjadi S Tomar AS Saffari A Fall CHD Prentice AM. Candidate Genes Linking Maternal Nutrient Exposure to Offspring Health via DNA Methylation: A Review of Existing Evidence in Humans With Specific Focus on One-Carbon Metabolism. Int J Epidemiol (2018) 47(6):1910–37. doi: 10.1093/ije/dyy153/5075855 PMC6280938 30137462

[76] Walsh CS Ogawa S Karahashi H Scoles DR Pavelka JC Tran H. ERCC5 Is a Novel Biomarker of Ovarian Cancer Prognosis. J Clin Oncol (2008) 26(18):2952–8. doi: 10.1200/JCO.2007.13.5806 18565881

[77] Zhu KP Zhang CL Ma XL Hu JP Cai T Zhang L. Analyzing the Interactions of mRNAs and ncRNAs to Predict Competing Endogenous RNA Networks in Osteosarcoma Chemo-Resistance. Mol Ther (2019) 27(3):518–30. doi: 10.1016/j.ymthe.2019.01.001 PMC6401193 30692017

[78] Sung HY Park AK Ju W Ahn J-H. Overexpression of Mucin 13 Due to Promoter Methylation Promotes Aggressive Behavior in Ovarian Cancer Cells. Yonsei Med J (2014) 55(5):1206–13. doi: 10.3349/ymj.2014.55.5.1206 PMC4108803 25048476

[79] Lee Y-H Kim JH Zhou H Kim BW Wong DT. Salivary Transcriptomic Biomarkers for Detection of Ovarian Cancer: For Serous Papillary Adenocarcinoma. J Mol Med (Berl) (2012) 90(4):427–34. doi: 10.1007/s00109-011-0829-0 22095100

[80] Qiu JJ Lin YY Ding JX Feng WW Jin HY Hua KQ. Long Non-Coding RNA ANRIL Predicts Poor Prognosis and Promotes Invasion/Metastasis in Serous Ovarian Cancer. Int J Oncol (2015) 46(6):2497–505. doi: 10.3892/ijo.2015.2943 25845387

[81] Xu X Zheng Z Jia L Suo S Liu B Shao T. Overexpression of SMARCA2 or CAMK2D Is Associated With Cisplatin Resistance in Human Epithelial Ovarian Cancer. Oncol Lett (2018) 16(3):3796–804. doi: 10.3892/ol.2018.9109 PMC6096159 30127991

[82] Gunel T Dogan B Gumusoglu E Hosseini MK Topuz S Aydinli K. Regulation of HMGA2 and KRAS Genes in Epithelial Ovarian Cancer by miRNA Hsa-Let-7d-3p. J Cancer Res Ther (2019) 15(6):1321–7. doi: 10.4103/jcrt.JCRT_866_18 31898667

[83] Resnick KE Alder H Hagan JP Richardson DL Croce CM Cohn DE. The Detection of Differentially Expressed microRNAs From the Serum of Ovarian Cancer Patients Using a Novel Real-Time PCR Platform. Gynecol Oncol (2009) 112(1):55–9. doi: 10.1016/j.ygyno.2008.08.036 18954897

[84] Nakamura N Terai Y Nunode M Kokunai K Konishi H Taga S. The Differential Expression of miRNAs Between Ovarian Endometrioma and Endometriosis-Associated Ovarian Cancer. J Ovarian Res (2020) 13(1):51. doi: 10.1186/s13048-020-00652-5 32359364 PMC7196233

[85] Günel T Gumusoglu E Dogan B Ertem FB Hosseini MK Cevik N. Potential Biomarker of Circulating hsa-miR-1273g-3p Level for Detection of Recurrent Epithelial Ovarian Cancer. Arch Gynecol Obstet (2018) 298(6):1173–80. doi: 10.1007/s00404-018-4913-3 30264202

[86] Chong GO Jeon H-S Han HS Son JW Lee YH Hong DG. Overexpression of microRNA-196b Accelerates Invasiveness of Cancer Cells in Recurrent Epithelial Ovarian Cancer Through Regulation of Homeobox A9. Cancer Genomics Proteomics (2017) 14(2):137–41. doi: 10.21873/cgp.20026 PMC5369313 28387653

[87] Chong GO Jeon H-S Han HS Son JW Lee YH Hong DG. Differential MicroRNA Expression Profiles in Primary and Recurrent Epithelial Ovarian Cancer. Anticancer Res (2015) 35(5):2611–7. 25964536

[88] Petrillo M Zannoni GF Beltrame L Martinelli E DiFeo A Paracchini L. Identification of High-Grade Serous Ovarian Cancer miRNA Species Associated With Survival and Drug Response in Patients Receiving Neoadjuvant Chemotherapy: A Retrospective Longitudinal Analysis Using Matched Tumor Biopsies. Ann Oncol (2016) 27(4):625–34. doi: 10.1093/annonc/mdw007 26782955

[89] Marzec-Kotarska B Cybulski M Kotarski JC Ronowicz A Tarkowski R Polak G. Molecular Bases of Aberrant miR-182 Expression in Ovarian Cancer. Genes Chromosomes Cancer (2016) 55(11):877–89. doi: 10.1002/gcc.22387 27295517

[90] Chung Y-W Bae H-S Song J-Y Lee JK Lee NW Kim T. Detection of microRNA as Novel Biomarkers of Epithelial Ovarian Cancer From the Serum of Ovarian Cancer Patients. Int J Gynecol Cancer (2013) 23(4):673–9. doi: 10.1097/IGC.0b013e31828c166d 23542579

[91] Li Y Yao L Liu F Hong J Chen L Zhang B. Characterization of microRNA Expression in Serous Ovarian Carcinoma. Int J Mol Med (2014) 34(2):491–8. doi: 10.3892/ijmm.2014.1813 24939816

[92] Li J Liang S Yu H Zhang J Ma D Lu X. An Inhibitory Effect of miR-22 on Cell Migration and Invasion in Ovarian Cancer. Gynecol Oncol (2010) 119(3):543–8. doi: 10.1016/j.ygyno.2010.08.034 20869762

[93] Nam EJ Yoon H Kim SW Kim H Kim YT Kim JH. MicroRNA Expression Profiles in Serous Ovarian Carcinoma. Clin Cancer Res (2008) 14(9):2690–5. doi: 10.1158/1078-0432.CCR-07-1731 18451233

[94] Shih KK Qin L-X Tanner EJ Zhou Q Bisogna M Dao F. A microRNA Survival Signature (MiSS) for Advanced Ovarian Cancer. Gynecol Oncol (2011) 121(3):444–50. doi: 10.1016/j.ygyno.2011.01.025 21354599

[95] Zhang L Volinia S Bonome T Calin GA Greshock J Yang N. Genomic and Epigenetic Alterations Deregulate microRNA Expression in Human Epithelial Ovarian Cancer. Proc Natl Acad Sci USA (2008) 105(19):7004–9. doi: 10.1073/pnas.0801615105 PMC2383982 18458333

[96] Prahm KP Høgdall C Karlsen MA Christensen IJ Novotny GW Høgdall E. Identification and Validation of Potential Prognostic and Predictive miRNAs of Epithelial Ovarian Cancer. PloS One (2018) 13(11):e0207319. doi: 10.1371/journal.pone.0207319 30475821 PMC6261038

[97] Ying HC Xu HY Lv J Ying TS Yang Q. MicroRNA Signatures of Platinum-Resistance in Ovarian Cancer. Eur J Gynaecol Oncol (2015) 36(1):16–20. 25872328

[98] Kazmierczak D Jopek K Sterzynska K Ginter-Matuszewska B Nowicki M Rucinski M. The Significance of MicroRNAs Expression in Regulation of Extracellular Matrix and Other Drug Resistant Genes in Drug Resistant Ovarian Cancer Cell Lines. Int J Mol Sci (2020) 21(7):2619. doi: 10.3390/ijms21072619 PMC7177408 32283808

[99] Liu R Guo H Lu S. MiR-335-5p Restores Cisplatin Sensitivity in Ovarian Cancer Cells Through Targeting BCL2L2. Cancer Med (2018) 7(9):4598–609. doi: 10.1002/cam4.1682 PMC6143943 30019389

[100] Li N Yang L Wang H Yi T Jia X Chen C. MiR-130a and MiR-374a Function as Novel Regulators of Cisplatin Resistance in Human Ovarian Cancer A2780 Cells. PloS One (2015) 10(6):e0128886. doi: 10.1371/journal.pone.0128886 26043084 PMC4456206

[101] Sorrentino A Liu C-G Addario A Peschle C Scambia G Ferlini C. Role of microRNAs in Drug-Resistant Ovarian Cancer Cells. Gynecol Oncol (2008) 111(3):478–86. doi: 10.1016/j.ygyno.2008.08.017 18823650

[102] Li X Lu Y Chen Y Lu W Xie X. MicroRNA Profile of Paclitaxel-Resistant Serous Ovarian Carcinoma Based on Formalin-Fixed Paraffin-Embedded Samples. BMC Cancer (2013) 13:216. doi: 10.1186/1471-2407-13-216 23627607 PMC3648441

[103] Shuang T Shi C Chang S Wang M Bai CH. Downregulation of miR-17~92 Expression Increase Paclitaxel Sensitivity in Human Ovarian Carcinoma SKOV3-TR30 Cells *via* BIM Instead of PTEN. Int J Mol Sci (2013) 14(2):3802–16. doi: 10.3390/ijms14023802 PMC3588071 23396109

[104] Huh JH Kim TH Kim K Song J-A Jung YJ Jeong J-Y. Dysregulation of miR-106a and miR-591 Confers Paclitaxel Resistance to Ovarian Cancer. Br J Cancer (2013) 109(2):452–61. doi: 10.1038/bjc.2013.305 PMC3721386 23807165

[105] Zhou Y Wang M Wu J Jie Z Chang S Shuang T. The Clinicopathological Significance of miR-1307 in Chemotherapy Resistant Epithelial Ovarian Cancer. J Ovarian Res (2015) 8:23. doi: 10.1186/s13048-015-0143-5 25887170 PMC4449560

[106] Yang N Kaur S Volinia S Greshock J Lassus H Hasegawa K. MicroRNA Microarray Identifies Let-7i as a Novel Biomarker and Therapeutic Target in Human Epithelial Ovarian Cancer. Cancer Res (2008) 68(24):10307–14. doi: 10.1158/0008-5472.CAN-08-1954 PMC2762326 19074899

[107] Chan JK Blansit K Kiet T Sherman A Wong G Earle C. The Inhibition of miR-21 Promotes Apoptosis and Chemosensitivity in Ovarian Cancer. Gynecol Oncol (2014) 132(3):739–44. doi: 10.1016/j.ygyno.2014.01.034 24472409

[108] Kobayashi M Sawada K Nakamura K Yoshimura A Miyamoto M Shimizu A. Exosomal miR-1290 Is a Potential Biomarker of High-Grade Serous Ovarian Carcinoma and Can Discriminate Patients From Those With Malignancies of Other Histological Types. J Ovarian Res (2018) 11(1):81. doi: 10.1186/s13048-018-0458-0 30219071 PMC6138886

[109] Yoshimura A Sawada K Nakamura K Kinose Y Nakatsuka E Kobayashi M. Exosomal miR-99a-5p Is Elevated in Sera of Ovarian Cancer Patients and Promotes Cancer Cell Invasion by Increasing Fibronectin and Vitronectin Expression in Neighboring Peritoneal Mesothelial Cells. BMC Cancer (2018) 18(1):1065. doi: 10.1186/s12885-018-4974-5 30396333 PMC6217763

[110] Liu S Du Q Rao Y Liu C Qu P. Long Non-Coding RNA NPBWR1-2 Affects the Development of Ovarian Cancer *via* Multiple microRNAs. Oncol Lett (2020) 20(1):685–92. doi: 10.3892/ol.2020.11639 PMC7285903 32565993

[111] Wang L He M Fu L Jin Y. Role of LncRNAHCP5/microRNA-525-5p/PRC1 Crosstalk in the Malignant Behaviors of Ovarian Cancer Cells. Exp Cell Res (2020) 5:112129. doi: 10.1016/j.yexcr.2020.112129 32511950

[112] Ni H Niu L-L Tian S-C Jing L-K Zhang L-T Lin Q-Q. Long Non-Coding RNA LINC00152 Is Up-Regulated in Ovarian Cancer Tissues and Regulates Proliferation and Cell Cycle of SKOV3 Cells. Eur Rev Med Pharmacol Sci (2019) 23(22):9803–13. doi: 10.26355/eurrev_201911_19543 31799647

[113] Lu Y-M Wang Y Liu S-Q Zhou M-Y Guo Y-R. Profile and Validation of Dysregulated Long Non−Coding RNAs and mRNAs in Ovarian Cancer. Oncol Rep (2018) 40(5):2964–76. doi: 10.3892/or.2018.6654 30132558

[114] Wang J Ding W Xu Y Tao E Mo M Xu W. Long Non-Coding RNA RHPN1-AS1 Promotes Tumorigenesis and Metastasis of Ovarian Cancer by Acting as a ceRNA Against miR-596 and Upregulating Letm1. Aging (Albany NY) (2020) 12(5):4558–72. doi: 10.18632/aging.102911 PMC7093190 32163372

[115] Tian X Song J Zhang X Yan M Wang S Wang Y. MYC-Regulated Pseudogene HMGA1P6 Promotes Ovarian Cancer Malignancy *via* Augmenting the Oncogenic HMGA1/2. Cell Death Dis (2020) 11(3):167. doi: 10.1038/s41419-020-2356-9 32127525 PMC7054391

[116] Guo Q Wang L Zhu L Lu X Song Y Sun J. The Clinical Significance and Biological Function of lncRNA SOCAR in Serous Ovarian Carcinoma. Gene (2019) 713:143969. doi: 10.1016/j.gene.2019.143969 31299360

[117] Yang Y Zhang H Xie Y Zhang S Zhu J Yin G. Preliminary Screening and Identification of Differentially Expressed Metastasis-Related ncRNAs in Ovarian Cancer. Oncol Lett (2018) 15(1):368–74. doi: 10.3892/ol.2017.7338 PMC5769367 29387224

[118] Zhu L Guo Q Lu X Zhao J Shi J Wang Z. CTD-2020K17.1, a Novel Long Non-Coding RNA, Promotes Migration, Invasion, and Proliferation of Serous Ovarian Cancer Cells *In Vitro* . Med Sci Monit (2018) 24:1329–39. doi: 10.12659/MSM.908456 PMC5848717 29504606

[119] Liu S-P Yang J-X Cao D-Y Shen K. Identification of Differentially Expressed Long Non-Coding RNAs in Human Ovarian Cancer Cells With Different Metastatic Potentials. Cancer Biol Med (2013) 10(3):138–41. doi: 10.7497/j.issn.2095-3941.2013.03.003 PMC3860336 24379988

[120] Qiu J Ye L Ding J Feng W Zhang Y Lv T. Effects of Oestrogen on Long Noncoding RNA Expression in Oestrogen Receptor Alpha-Positive Ovarian Cancer Cells. J Steroid Biochem Mol Biol (2014) 141:60–70. doi: 10.1016/j.jsbmb.2013.12.017 24380700

[121] Shi C Wang M. LINC01118 Modulates Paclitaxel Resistance of Epithelial Ovarian Cancer by Regulating miR-134/Abcc1. Med Sci Monit (2018) 24:8831–9. doi: 10.12659/msm.910932 PMC6292151 30521500

[122] Horita K Kurosaki H Nakatake M Kuwano N Oishi T Itamochi H. lncRNA UCA1-Mediated Cdc42 Signaling Promotes Oncolytic Vaccinia Virus Cell-To-Cell Spread in Ovarian Cancer. Mol Ther Oncolytics (2019) 13:35–48. doi: 10.1016/j.omto.2019.03.003 31011626 PMC6463205

[123] Li Z Niu H Qin Q Yang S Wang Q Yu C. lncRNA UCA1 Mediates Resistance to Cisplatin by Regulating the miR-143/FOSL2-Signaling Pathway in Ovarian Cancer. Mol Ther Nucleic Acids (2019) 17:92–101. doi: 10.1016/j.omtn.2019.05.007 31234009 PMC6595407

[124] Xu M Zhou K Wu Y Wang L Lu S. Linc00161 Regulated the Drug Resistance of Ovarian Cancer by Sponging microRNA-128 and Modulating Mapk1. Mol Carcinog (2019) 58(4):577–87. doi: 10.1002/mc.22952 30556928

[125] Yan H Xia JY Feng FZ. Long Non-Coding RNA ENST00000457645 Reverses Cisplatin Resistance in CP70 Ovarian Cancer Cells. Genet Mol Res (2017) 16(1). doi: 10.4238/gmr16019411 28128423

[126] Long X Song K Hu H Tian Q Wang W Dong Q. Long Non-Coding RNA GAS5 Inhibits DDP-Resistance and Tumor Progression of Epithelial Ovarian Cancer *via* GAS5-E2F4-PARP1-MAPK Axis. J Exp Clin Cancer Res (2019) 38(1):345. doi: 10.1186/s13046-019-1329-2 31391118 PMC6686414

[127] Gao Y Zhang C Liu Y Wang M. Circular RNA Profiling Reveals CircRNA1656 as a Novel Biomarker in High Grade Serous Ovarian Cancer. Biosci Trends (2019) 13(2):204–11. doi: 10.5582/bst.2019.01021 31019161

[128] Ning L Long B Zhang W Yu M Wang S Cao D. Circular RNA Profiling Reveals Circexoc6b and Circn4bp2l2 as Novel Prognostic Biomarkers in Epithelial Ovarian Cancer. Int J Oncol (2018) 53(6):2637–46. doi: 10.3892/ijo.2018.4566 30272264

[129] Zhang S Cheng J Quan C Wen H Feng Z Hu Q. circCELSR1 (Hsa_Circ_0063809) Contributes to Paclitaxel Resistance of Ovarian Cancer Cells by Regulating FOXR2 Expression *via* miR-1252. Mol Ther Nucleic Acids (2020) 19:718–30. doi: 10.1016/j.omtn.2019.12.005 PMC6965731 31945729

[130] Zhao Z Ji M Wang Q He N Li Y. Circular RNA Cdr1as Upregulates SCAI to Suppress Cisplatin Resistance in Ovarian Cancer via miR-1270 Suppression. Mol Ther Nucleic Acids (2019) 18:24–33. doi: 10.1016/j.omtn.2019.07.012 31479922 PMC6726918

[131] Eoh KJ Lee SH Kim HJ Lee J-Y Kim S Kim SW. MicroRNA-630 Inhibitor Sensitizes Chemoresistant Ovarian Cancer to Chemotherapy by Enhancing Apoptosis. Biochem Biophys Res Commun (2018) 497(2):513–20. doi: 10.1016/j.bbrc.2018.02.062 29452092

[132] Kuhlmann JD Baraniskin A Hahn SA Mosel F Bredemeier M Wimberger P. Circulating U2 Small Nuclear RNA Fragments as a Novel Diagnostic Tool for Patients With Epithelial Ovarian Cancer. Clin Chem (2014) 60(1):206–13. doi: 10.1373/clinchem.2013.213066 24212085

[133] He A He S Li X Zhou L. ZFAS1: A Novel Vital Oncogenic lncRNA in Multiple Human Cancers. Cell Prolif (2019) 52(1):e12513. doi: 10.1111/cpr.12513 30288832 PMC6430496

[134] Liu S Jiang X Li W Cao D Shen K Yang J. Inhibition of the Long Non-Coding RNA MALAT1 Suppresses Tumorigenicity and Induces Apoptosis in the Human Ovarian Cancer SKOV3 Cell Line. Oncol Lett (2016) 11(6):3686–92. doi: 10.3892/ol.2016.4435 PMC4888020 27313681

[135] Lasda E Parker R. Circular RNAs: Diversity of Form and Function. RNA (2014) 20(12):1829–42. doi: 10.1261/rna.047126.114 PMC4238349 25404635

[136] Wang Y Lu T Wang Q Liu J Jiao W. Circular RNAs: Crucial Regulators in the Human Body (Review). Oncol Rep (2018) 40(6):3119–35. doi: 10.3892/or.2018.6733 PMC6196641 30272328

[137] Cheng Y Liu C Zhang N Wang S Zhang Z. Proteomics Analysis for Finding Serum Markers of Ovarian Cancer. BioMed Res Int (2014) 2014:179040. doi: 10.1155/2014/179040 25250314 PMC4164372

[138] Shetty V Hafner J Shah P Nickens Z Philip R. Investigation of Ovarian Cancer Associated Sialylation Changes in N-Linked Glycopeptides by Quantitative Proteomics. Clin Proteomics (2012) 9(1):10. doi: 10.1186/1559-0275-9-10 22856521 PMC3488482

[139] Abbott KL Lim J-M Wells L Benigno BB McDonald JF Pierce M. Identification of Candidate Biomarkers With Cancer-Specific Glycosylation in the Tissue and Serum of Endometrioid Ovarian Cancer Patients by Glycoproteomic Analysis. Proteomics (2010) 10(3):470–81. doi: 10.1002/pmic.200900537 PMC4932840 19953551

[140] Swiatly A Horala A Matysiak J Hajduk J Nowak-Markwitz E Kokot ZJ. Understanding Ovarian Cancer: iTRAQ-Based Proteomics for Biomarker Discovery. Int J Mol Sci (2018) 19(8):2240. doi: 10.3390/ijms19082240 PMC6121953 30065196

[141] Kim SI Jung M Dan K Lee S Lee C Kim HS. Proteomic Discovery of Biomarkers to Predict Prognosis of High-Grade Serous Ovarian Carcinoma. Cancers (Basel) (2020) 12(4):790. doi: 10.3390/cancers12040790 PMC7226362 32224886

[142] Zhang F Li C Liu H Wang Y Chen Y Wu X. The Functional Proteomics Analysis of VEGF-Treated Human Epithelial Ovarian Cancer Cells. Tumour Biol (2014) 35(12):12379–87. doi: 10.1007/s13277-014-2552-2 25192722

[143] Zhang X Feng Y Wang XY Zhang YN Yuan CN Zhang SF. The Inhibition of UBC13 Expression and Blockage of the DNMT1-CHFR-Aurora A Pathway Contribute to Paclitaxel Resistance in Ovarian Cancer. Cell Death Dis (2018) 9(2):93. doi: 10.1038/s41419-017-0137-x 29367628 PMC5833742

[144] Wang Y Li H. Identification of Proteins Associated With Paclitaxel Resistance of Epithelial Ovarian Cancer Using iTRAQ-Based Proteomics. Oncol Lett (2018) 15(6):9793–801. doi: 10.3892/ol.2018.8600 PMC6004651 29928353

[145] Di Michele M Marcone S Cicchillitti L Della Corte A Ferlini C Scambia G. Glycoproteomics of Paclitaxel Resistance in Human Epithelial Ovarian Cancer Cell Lines: Towards the Identification of Putative Biomarkers. J Proteomics (2010) 73(5):879–98. doi: 10.1016/j.jprot.2009.11.012 19951750

[146] Bateman NW Jaworski E Ao W Wang G Litzi T Dubil E. Elevated AKAP12 in Paclitaxel-Resistant Serous Ovarian Cancer Cells Is Prognostic and Predictive of Poor Survival in Patients. J Proteome Res (2015) 14(4):1900–10. doi: 10.1021/pr5012894 PMC4612617 25748058

[147] Avril S Dincer Y Malinowsky K Wolff C Gündisch S Hapfelmeier A. Increased PDGFR-Beta and VEGFR-2 Protein Levels Are Associated With Resistance to Platinum-Based Chemotherapy and Adverse Outcome of Ovarian Cancer Patients. Oncotarget (2017) 8(58):97851–61. doi: 10.18632/oncotarget.18415 PMC5716696 29228656

[148] Zhang Z Qin K Zhang W Yang B Zhao C Zhang X. Postoperative Recurrence of Epithelial Ovarian Cancer Patients and Chemoresistance Related Protein Analyses. J Ovarian Res (2019) 12(1):29. doi: 10.1186/s13048-019-0499-z 30917846 PMC6436226

[149] Cruz IN Coley HM Kramer HB Madhuri TK Safuwan NA Angelino AR. Proteomics Analysis of Ovarian Cancer Cell Lines and Tissues Reveals Drug Resistance-Associated Proteins. Cancer Genomics Proteomics (2017) 14(1):35–51. doi: 10.21873/cgp.20017 28031236 PMC5267499

[150] Huang X Zhou J Tang R Han S Zhou X. Potential Significance of Peptidome in Human Ovarian Cancer for Patients With Ascites. Int J Gynecol Cancer (2018) 28(2):355–62. doi: 10.1097/IGC.0000000000001166 29240604

[151] Zhang S-F Wang X-Y Fu Z-Q Peng Q-H Zhang J-Y Ye F. TXNDC17 Promotes Paclitaxel Resistance *via* Inducing Autophagy in Ovarian Cancer. Autophagy (2015) 11(2):225–38. doi: 10.1080/15548627.2014.998931 PMC4502659 25607466

[152] Jin Z Kang J Yu T. Missing Value Imputation for LC-MS Metabolomics Data by Incorporating Metabolic Network and Adduct Ion Relations. Bioinformatics (2018) 34(9):1555–61. doi: 10.1093/bioinformatics/btx816 PMC5925787 29272352

[153] Yang W Mu T Jiang J Sun Q Hou X Sun Y. Identification of Potential Biomarkers and Metabolic Profiling of Serum in Ovarian Cancer Patients Using UPLC/Q-TOF MS. Cell Physiol Biochem (2018) 51(3):1134–48. doi: 10.1159/000495492 30476914

[154] Plewa S Horała A Dereziński P Nowak-Markwitz E Matysiak J Kokot ZJ. Wide Spectrum Targeted Metabolomics Identifies Potential Ovarian Cancer Biomarkers. Life Sci (2019) 222:235–44. doi: 10.1016/j.lfs.2019.03.004 30853626

[155] Yin R Yang T Su H Ying L Liu L Sun C. Saturated Fatty Acids as Possible Important Metabolites for Epithelial Ovarian Cancer Based on the Free and Esterified Fatty Acid Profiles Determined by GC-MS Analysis. Cancer biomark (2016) 17(3):259–69. doi: 10.3233/CBM-160638 PMC13020504 27802202

[156] Cheng Y Li L Zhu B Liu F Wang Y Gu X. Expanded Metabolomics Approach to Profiling Endogenous Carbohydrates in the Serum of Ovarian Cancer Patients. J Sep Sci (2016) 39(2):316–23. doi: 10.1002/jssc.201500964 26549419

[157] Chen J Zhang X Cao R Lu X Zhao S Fekete A. Serum 27-Nor-5β-Cholestane-3,7,12,24,25 Pentol Glucuronide Discovered by Metabolomics as Potential Diagnostic Biomarker for Epithelium Ovarian Cancer. J Proteome Res (2011) 10(5):2625–32. doi: 10.1021/pr200173q 21456628

[158] Ke C Hou Y Zhang H Fan L Ge T Guo B. Large-Scale Profiling of Metabolic Dysregulation in Ovarian Cancer. Int J Cancer (2015) 136(3):516–26. doi: 10.1002/ijc.29010 24895217

[159] Fan L Yin M Ke C Ge T Zhang G Zhang W. Use of Plasma Metabolomics to Identify Diagnostic Biomarkers for Early Stage Epithelial Ovarian Cancer. J Cancer (2016) 7(10):1265–72. doi: 10.7150/jca.15074 PMC4934035 27390602

[160] Zhang F Zhang Y Ke C Li A Wang W Yang K. Predicting Ovarian Cancer Recurrence by Plasma Metabolic Profiles Before and After Surgery. Metabolomics (2018) 14(5):65. doi: 10.1007/s11306-018-1354-8 30830339

[161] Zhang T Wu X Ke C Yin M Li Z Fan L. Identification of Potential Biomarkers for Ovarian Cancer by Urinary Metabolomic Profiling. J Proteome Res (2013) 12(1):505–12. doi: 10.1021/pr3009572 23163809

[162] Chen J Zhou L Zhang X Lu X Cao R Xu C. Urinary Hydrophilic and Hydrophobic Metabolic Profiling Based on Liquid Chromatography-Mass Spectrometry Methods: Differential Metabolite Discovery Specific to Ovarian Cancer. Electrophoresis (2012) 33(22):3361–9. doi: 10.1002/elps.201200140 23109122

[163] Buckendahl A-C Budczies J Fiehn O Darb-Esfahani S Kind T Noske A. Prognostic Impact of AMP-Activated Protein Kinase Expression in Ovarian Carcinoma: Correlation of Protein Expression and GC/TOF-MS-Based Metabolomics. Oncol Rep (2011) 25(4):1005–12. doi: 10.3892/or.2011.1162 21271224

[164] Motamedian E Ghavami G Sardari S. Investigation on Metabolism of Cisplatin Resistant Ovarian Cancer Using a Genome Scale Metabolic Model and Microarray Data. Iran J Basic Med Sci (2015) 18(3):267–76. PMC4414993 25945240

